# Genome-wide annotation and characterization of CLAVATA/ESR (CLE) peptide hormones of soybean (*Glycine max*) and common bean (*Phaseolus vulgaris*), and their orthologues of *Arabidopsis thaliana*

**DOI:** 10.1093/jxb/erv351

**Published:** 2015-07-17

**Authors:** April H. Hastwell, Peter M. Gresshoff, Brett J. Ferguson

**Affiliations:** Centre for Integrative Legume Research, School of Agricultural and Food Sciences, The University of Queensland, St Lucia, Brisbane, Queensland, 4072, Australia

**Keywords:** Autoregulation of nodulation, nitrate regulation of nodulation, plant development, plant hormone, plant peptide signalling, symbiosis

## Abstract

CLE peptides are key regulators of cell proliferation and differentiation in plant shoots, roots, vasculature, and legume nodules. They are C-terminally encoded peptides that are post-translationally cleaved and modified from their corresponding pre-propeptides to produce a final ligand that is 12–13 amino acids in length. In this study, an array of bionformatic and comparative genomic approaches was used to identify and characterize the complete family of CLE peptide-encoding genes in two of the world’s most important crop species, soybean and common bean. In total, there are 84 CLE peptide-encoding genes in soybean (considerably more than the 32 present in *Arabidopsis*), including three pseudogenes and two multi-CLE domain genes having six putative CLE domains each. In addition, 44 CLE peptide-encoding genes were identified in common bean. *In silico* characterization was used to establish all soybean homeologous pairs, and to identify corresponding gene orthologues present in common bean and *Arabidopsis*. The soybean CLE pre-propeptide family was further analysed and separated into seven distinct groups based on structure, with groupings strongly associated with the CLE domain sequence and function. These groups provide evolutionary insight into the CLE peptide families of soybean, common bean, and *Arabidopsis*, and represent a novel tool that can aid in the functional characterization of the peptides. Transcriptional evidence was also used to provide further insight into the location and function of all CLE peptide-encoding members currently available in gene atlases for the three species. Taken together, this in-depth analysis helped to identify and categorize the complete CLE peptide families of soybean and common bean, established gene orthologues within the two legume species, and *Arabidopsis*, and provided a platform to help compare, contrast, and identify the function of critical CLE peptide hormones in plant development.

## Highlight

Using a genome-wide approach, the complete CLE peptide-encoding gene families of soybean and common bean were identified, characterized, and compared with those of *Arabidopsis*.

## Introduction

CLAVATA/embryo surrounding region (ESR) peptide hormones (CLE peptides) are a group of post-translationally modified signal molecules involved in the regulation and differentiation of meristematic plant tissues. They have been shown to control cell divisions in the shoot apical meristem (SAM), root apical meristem (RAM), vasculature, and legume nodules (Matsubayashi, 2014; Ferguson and Mathesius, 2014; Grienenberger and Fletcher, 2015; Hastwell *et al.*, 2015). They arise from a structurally conserved gene family and are named after the first identified CLE peptide (AtCLV3 in *Arabidopsis thaliana*; Fletcher *et al.*, 1999), and the structurally and functionally similar, but unrelated, ESR peptides (first identified in *Zea mays*; Opsahl-Ferstad *et al.*, 1997; Cock and McCormick, 2001).

Mature CLE peptides are typically 12–13 amino acids in length and are located at or near the C-terminus of their pre-propeptide. CLE pre-propeptides are cysteine-poor and have a tripartite domain structure, consisting of an N-terminal signal peptide, a central variable domain, and a highly conserved and functional CLE peptide domain (Matsubayashi, 2014; Hastwell *et al.*, 2015). Some also have a fourth domain, called a C-terminal extension, which is not highly conserved, except between orthologous genes. Multi-CLE domain-containing pre-propeptides have also been identified in several plant species (Kinoshita *et al.*, 2007; Oelkers *et al.*, 2008), but little is known about their processing in plants. There is also a group of CLE-Like (CLEL) peptides, whose functional domain shares a similar structure but exhibits unrelated activity (Meng *et al.*, 2012). Interestingly, one gene identified in *Arabidopsis* (*AtCLE18*) contains both a CLE and a CLEL domain (Meng *et al.*, 2012).

The mature CLE peptide ligand is post-translationally cleaved and modified from its pre-propeptide. Hydroxylatation of proline residues is common, with one central hydroxyproline having a tri-arabinose moiety attached (Matsubayashi, 2014); however, it is important to note that all arabinose post-translational modifications identified in plants to date are limited to three peptides in *A. thaliana* (AtCLV3, AtCLE2, and AtCLE9) and one in *Lotus japonicus* (LjCLE-RS2) (Ohyama *et al.*, 2009; Okamoto *et al.*, 2013; Shinohara and Matsubayashi, 2013; Matsubayashi, 2014). Mature CLE peptides are ligands for leucine-rich repeat receptor kinases (LRR-RKs), with the first identified ligand receptor pair being CLV3 and CLV1 of *Arabidopsis* (Fletcher *et al.*, 1999), which has since expanded to include a number of additional binding partners and associated factors (Shinohara and Matsubayashi, 2015). A comprehensive list of putative CLE ligand–LRR-RK pairs was recently presented (Endo *et al.*, 2014).

The role of many CLE peptides remains unknown, with the majority that have been functionally characterized found in *Arabidopsis*. The most widely studied is AtCLV3, which acts in the SAM to regulate stem cell numbers (Fletcher *et al.*, 1999; Gaillochet *et al.*, 2015). Additional *Arabidopsis* CLE peptides acting in the root have also been characterized, including AtCLE40 (Hobe *et al.*, 2003; Sharma *et al.*, 2003; Stahl *et al.*, 2009), which regulates cell proliferation in the RAM as part of a mechanism mirroring that acting in the SAM (van der Graff *et al.*, 2009). Other root-acting CLE peptides of *Arabidopsis* include AtCLE1, 2, 3, 4, and 7, which are involved in nitrate-responsive mechanisms, with some also involved in lateral root development (Scheible *et al.*, 2004; Araya *et al.*, 2014). Additional CLE peptide-encoding genes involved in cell proliferation and differentiation include *AtCLE8*, which acts in embryogenesis (Fiume and Fletcher, 2012), and *AtCLE45*, which has been implicated in both root protophloem and pollen development (Depuydt *et al.*, 2013; Endo *et al.*, 2013; Rodriguez-Villalon *et al.*, 2014). Three CLE peptides, known as tracheary element differentiation factors (TDIFs), control vascular meristematic tissue proliferation and differentiation (encoded by *AtCLE41*, *AtCLE42*, and *AtCLE44*; Sawa *et al.*, 2006; Ito *et al.*, 2006; Hirakawa *et al.*, 2010). This group has the highest conservation amongst gymnosperms and angiosperms (Strabala *et al.*, 2014), and consists of the only CLE peptides to begin with a histidine, rather than the archetypical arginine residue that is characteristic of all other CLE peptides (with the sole exception of AtCLE46, whose CLE domain begins with a histidine, and whose function remains unknown; Hirakawa *et al.*, 2011).

In addition to those identified in *Arabidopsis*, a number of CLE peptides have been identified in various legume species. This includes CLE peptides acting to control the highly important nodulation process, which is a symbiotic relationship legumes enter into with nitrogen-fixing rhizobia bacteria (Okamoto *et al.*, 2009, 2013; Mortier *et al.*, 2010, 2012; Reid *et al.*, 2011*a*, 2013; Ferguson *et al.*, 2014; reviewed in Hastwell *et al.*, 2015). By regulating nodulation, these CLE peptides essentially enable the host plant to balance nitrogen uptake from the bacteria with resource allocation to form and maintain nodules (Ferguson *et al.*, 2010). Prominent pathways involved in this regulation are the systemic autoregulation of nodulation (AON) and the local nitrogen regulation pathways, both of which commence with the induction of CLE peptide signals (reviewed in Ferguson *et al.*, 2010; Reid *et al.*, 2011*b*). Similarly, a number of legume CLE peptides have also been shown to respond to phosphate application (Funayama-Noguchi *et al.*, 2011) and more recently mycorrhiza infection (Handa *et al.*, 2015).

Aside from plants, cyst nematodes are the only other known organism to have CLE peptide-encoding genes (Mitchum *et al.*, 2013). These genes have multiple CLE domains that are processed into a single mature peptide ligand (Chen *et al.*, 2015). The peptides are thought to assist in nematode infection, possibly by manipulating the host to gain entry into the plant (Olsen and Skriver, 2003; Wang *et al.*, 2005; reviewed in Mitchum *et al.*, 2013). They are post-translationally modified and processed by the host plant’s machinery, and are perceived by plant receptors (Replogle *et al.*, 2011; Chen *et al.*, 2015), suggesting that they may have evolved through horizontal gene transfer.

Here, advantage was taken of recent advances in genomics and bioinformatics to identify, categorize, and functionally characterize the highly important CLE peptide families of soybean and common bean, two agriculturally important crop species. Soybean and common bean share a common ancestor whose genome duplicated ~59 million years ago (MYA), from which soybean subsequently diverged (19 MYA) and duplicated again 13 MYA (Lavin *et al.*, 2005; Schmutz *et al.*, 2010, 2014). As a result, 75% of soybean genes have more than one copy across the genome (a homeologous or duplicate copy; Schmutz *et al.*, 2010, 2014; Roulin *et al.*, 2013), whereas common bean does not. Indeed, for these reasons, soybean and common bean are commonly used for comparative and evolutionary studies in genomics and genetics (e.g. McClean *et al.*, 2008; Lin *et al.*, 2010; Ferguson *et al.* 2014; Schmutz *et al.*, 2014).

The present investigations identified a total of 84 CLE peptide-encoding genes in soybean and 44 in common bean. In-depth sequence analyses enabled the identification of all homeologous copies within soybean, in addition to all orthologous copies existing between soybean, common bean, and *Arabidopsis*. Transcriptional analysis of all CLE peptide-encoding genes available in gene atlases of soybean, common bean, and *Arabidopsis* were evaluated to provide further insight into the localization and function of the genes. Moreover, using the complete family in soybean, seven distinct CLE peptide groups were defined based on both sequence similarity and phylogenetic analysis, with consensus sequences subsequently derived for each. Collectively, the findings provide new insight into the sequence, structure, and evolution of critical CLE peptide hormones of plants.

## Materials and methods

### Gene identification

To identify CLE peptide-encoding genes, multiple TBLASTN and BLASTN searches using known soybean sequences were conducted in Phytozome against the *Glycine max Wm82.a2.v1* and *Phaseolus vulgaris v1.0* genomes (http://www.phytozome.net/; Schmutz *et al.*, 2010, 2014; Goodstein *et al.*, 2012). Searches were conducted using less stringent parameters [expected threshold (E)=10] to enhance the identification of genes of interest. Results were then manually validated to confirm the presence of a CLE domain in an open reading frame. Subsequent searches based on the preliminary findings were performed using BLASTN to identify additional genes, including common bean orthologues and soybean duplicates, particularly where no duplicate/orthologue was identified in the initial queries. These subsequent searches were conducted using a slightly more stringent parameter of E=1. The open reading frames of homologous chromosome regions were also examined for potential unannotated or truncated duplicates. Additional BLASTP searches of mycorrhizal (http://genome.jgi.doe.gov/) and rhizobia genomes (Rhizobase; http://genome.microbedb.jp/rhizobase; Fujisawa *et al.*, 2014), using both whole CLE pre-propeptide sequences and also CLE domain consensus sequences from soybean, were also performed using very low stringency (E=100) to identify CLE peptide encoding genes in these species.

### Genomic environments

Synteny between genomic environments was individually obtained for each gene of interest. This was achieved using Phytozome JBrowse of the *Glycine max Wm82.a2.v1*, *Phaseolus vulgaris v1.0*, *Arabidopsis thaliana TAIR10*, *Oryza sativa v7.0* and *Medicago truncatula Mt4.0v1* genomes (http://www.phytozome.net/; Ouyang *et al.*, 2007; Schmutz *et al.*, 2010, 2014; Young *et al.*, 2011; Goodstein *et al.*, 2012; Lamesch *et al.*, 2012). For each genomic environment investigated, the five genes located directly up- and downstream of the gene of interest were assessed for their orientation, gene family, and predicted homologues.

### Sequence characterization

Clustal Omega, hosted on EMBL-EBI (http://www.ebi.ac.uk/Tools/msa/clustalo/), was used to generate multiple sequence alignments (Goujon *et al.*, 2010; Sievers *et al.*, 2011; McWilliam *et al.*, 2013). Manual adjustments were subsequently made to some of the sequences predicted in Phytozome, particularly in regards to their start codon. This was based on sequence similarity to duplicate genes, similarly clustering genes, and/or likely orthologous genes, in addition to signal peptide domain prediction results.

Logo diagrams used to define consensus sequences were obtained using multiple sequence alignments for each CLE peptide group (I–VII) in Geneious Pro v6.1.8 (Kearse *et al.*, 2012). Signal peptides were identified using the SignalP prediction program v4.1 (http://www.cbs.dtu.dk/services/SignalP/; Petersen *et al.*, 2011). Hydrophobicity values were determined from amino acid scale values on ProtScale (http://web.expasy.org/protscale/; Gasteiger *et al.*, 2005) using the Kyte and Doolittle (1982) hydrophobicity scale.

### Phylogenetic analyses

Phylogenetic trees were constructed from multiple sequence alignments using the PHYML plugin in Geneious Pro v6.1.8 (Guindon and Gascuel, 2003). They were derived using the maximum likelihood approach with 1000 bootstraps to support a branch, with the exception of the tree designed using all soybean, common bean, and *Arabidopsis* sequences, where 100 bootstraps were used. Multiple trees were constructed to identify homeologous soybean genes. Those appearing to lack a homeologous copy were identified and used to re-search the genome for a potential duplicate. All trees presented here include each distinct gene identified in the numerous searches made. A similar approach was used to identify all soybean gene orthologues in common bean and *Arabidopsis*.

### Meta-analyses of transcriptome data

Transcriptional data for the meta-analysis was collected from publicly available data sets from the Soybean RNA-Seq Atlas (http://www.soybase.org/soyseq/; Severin *et al.*, 2010); the Soybean eFP Browser (http://bar.utoronto.ca/efpsoybean/cgi-bin/efpWeb.cgi; Libault *et al.*, 2010*a*, b); A Common Bean Gene Expression Atlas (http://plantgrn.noble.org/PvGEA/index.jsp; Jamie *et al.* 2014); and the Arabidopsis eFP Browser (http://bar.utoronto.ca/efp/cgi-bin/efpWeb.cgi; Schmid *et al.*, 2005). The entire list of gene identifiers for each species was searched in their respective databases, and only those with transcriptional data are presented. Normalized RPKM (reads per kilobase per million) values were taken where possible.

## Results

### Identification of CLE peptide-encoding genes in soybean and common bean, in addition to mycorrhiza and rhizobia species

**Fig. 1.**
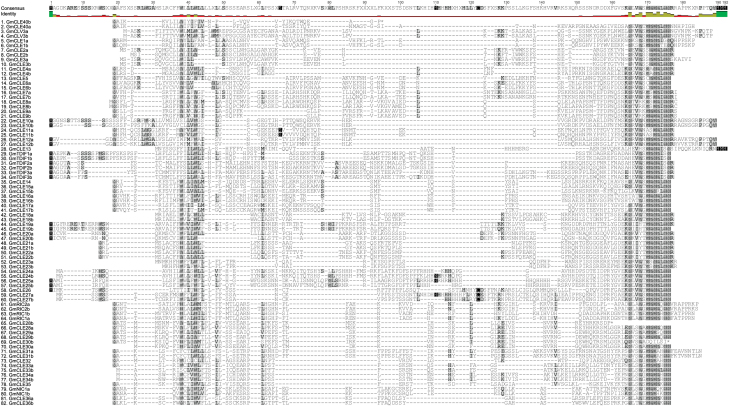
Multiple sequence alignment of soybean (*Glycine max*) CLE pre-propeptides. Homeologous copies consistently align together, as do other closely related sequences. Shading of amino acid residues represents conservation, with the darker the shading the more highly conserved the residues. The CLE domain and the leucine-rich region of the signal peptide domain exhibit the greatest degree of conservation across the entire pre-propeptide family. (*This figure is available in colour at JXB online.*)

**Fig. 2.**
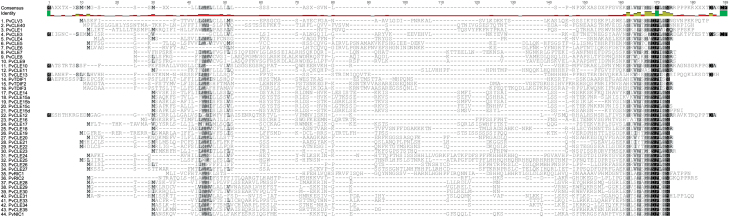
Multiple sequence alignment of common bean (*Phaseolus vulgaris*) CLE pre-propeptides. Related sequences tend to align closer together. Shading of amino acid residues represents conservation, with the darker the shading the more highly conserved the residues. As with the soybean prepropeptides shown in Fig. 1, the CLE domain and the leucine-rich region of the signal peptide domain exhibit the greatest degree of conservation across the entire pre-propeptide family. (*This figure is available in colour at JXB online*.)

To identify CLE peptide-encoding genes in soybean and common bean, a genome-wide analysis was performed involving multiple BLAST queries, followed by manual validation and the removal of false positives (i.e. no CLE domain). This resulted in the identification of 84 distinct soybean genes and 44 distinct common bean genes (Figs 1, 2; Tables 1, 2). BLAST queries were based on all known soybean CLE genes, and some *Arabidopsis* genes, and involved searching with both pre-propeptide and CLE domain sequences to enhance the likelihood of detecting all CLE peptide-encoding genes in the two genomes.

**Table 1.** *Features of the soybean* (Glycine max*) CLE genes*

Name	Chromosome location	Orientation	Pre-propeptide length^*a*^	Predicted intron	SP cleavage site^*b*^	Homeologue similarity (%)	Common bean orthologue	Soybean and common bean pairwise identity (%)
GmCLE1a	Chr11:10740675..10741635	Reverse	84	Y	23	82.1	PvCLE1	74.6
GmCLE1b	Chr12:4724973..4727049	Reverse	83	Y	23			
GmCLE2a	Chr20:46634836..46635799	Reverse	76	N	30	92.1	–	–
GmCLE2b	Chr10:38974407..38975417	Forward	74	N	28			
GmCLE3a	Chr03:43793053..43794104	Forward	81	N	27	89.5	PvCLE3	80.2
GmCLE3b	Chr19:48528559..48529545	Forward	75	N	27			
GmCLE4a	Chr01:53094482..53095085	Forward	67	N	21	92.5	PvCLE4	82.6
GmCLE4b	Chr11:3319115..3320325	Reverse	67	N	21			
GmCLE5	Chr08:46805591..46806636	Reverse	99	N	25	-	PvCLE5	69.9
GmCLE6a	Chr20:35756760..35757955	Reverse	97	N	26	91.8	PvCLE6	76.3
GmCLE6b	Chr10:49704427..49706416	Forward	96	N	26			
GmCLE7a	Chr01:5559528..5560353	Forward	108	N	23	89.8	PvCLE7	85.8
GmCLE7b	Chr02:10245905..10246706	Reverse	108	N	23			
GmCLE8a	Chr06:17294801..17295629	Reverse	96	N	21	83.9	PvCLE8	85.4
GmCLE8b	Chr04:42380768..42381923	Forward	95	N	28			
GmCLE9a	Chr05:2299498..2299782	Forward	79	Y	19	93.8	PvCLE9	80.3
GmCLE9b	Chr17:7902958..7904070	Reverse	79	Y	19			
GmCLE10a	Chr01:4182744..4185349	Reverse	108	Y	42	83.3	PvCLE10	79.7
GmCLE10b	Chr02:2311001..2311717	Forward	102	Y	40			
GmCLE11a	Chr14:7781256..7782013	Reverse	82	N	27	89.3	PvCLE11	65.4
GmCLE11b	Chr17:39269471..39270222	Forward	84	N	27			
GmCLE12a	Chr13:16671710..16673786	Forward	97	Y	34	94.8	PvCLE12	93.1
GmCLE12b	Chr19:1819967..1821863	Rreverse	97	Y	34			
GmCLE13	Chr13:36676213..36676962	Forward	86	Y	24	–	PvCLE13	73.8
GmCLE14	Chr10:46589943..46590137	Forward	83	N	25	–	PvCLE14	72.7
GmCLE15a	Chr10:46586624..46587350	Forward	86	N	25	51.1	PvCLE15a, PvCLE15b, PvCLE15c, PvCLE15d	48.3, 47.9, 45.4, 45.6
GmCLE15b	Chr06:27528956..27529216	Forward	86	N	26			
GmCLE16a	Chr09:34804635..34806006	Forward	86	N	27	90.7	PvCLE16	85.7
GmCLE16b	Chr16:35643819..35644747	Forward	86	N	27			
GmCLE17a	Chr05:38846465..38847260	Reverse	87	N	28	86.2	PvCLE17	85.1
GmCLE17b	Chr08:969117..970012	Reverse	87	N	24			
GmCLE18a	Chr13:21801637..21802409	Forward	85	N	19	85.9	PvCLE18	80.8
GmCLE18b	Chr17:4258185..4258436	Reverse	83	N	19			
GmCLE19a	Chr07:39333907..39334972	Forward	119	N	32	83.9	PvCLE19	67.0
GmCLE19b	Chr20:1750676..1751787	Forward	114	N	32			
GmCLE20a	Chr03:33954213..33955592	Forward	100	N	36	91.0	PvCLE20	78.9
GmCLE20b	Chr19:38764138..38765477	Forward	94	N	31			
GmCLE21a	Chr02:46067116..46071548	Forward	81	N	26	88.9	PvCLE21	75.4
GmCLE21b	Chr14:2730030..2731670	Reverse	80	N	26			
GmCLE22a	Chr07:41652868..41653137	Reverse	89	N	27	91.0	PvCLE22	74.0
GmCLE22b	Chr20:7721313..7721576	Reverse	87	N	27			
GmCLE23a	Chr02:45459965..45460989	Reverse	73	N	23	85.9	PvCLE23	79.0
GmCLE23b	Chr14:3533265..3534446	Forward	71	N	21			
GmCLE24a	Chr10:43660111..43661108	Forward	110	N	23	88.4	PvCLE24	82.9
GmCLE24b	Chr20:42379994..42380805	Reverse	111	N	23			
GmCLE25a	Chr05:1295698..1296578	Forward	118	N	29	80.0	PvCLE25	68.8
GmCLE25b	Chr17:9746590..9748712	Forward	114	N	29			
GmCLE26	Chr20:2984627..2986271	Forward	99	N	27	–	PvCLE26	52.6
GmCLE27a	Chr02:11156483..11156827	Reverse	114	N	30	83.3	PvCLE27	78.6
GmCLE27b	Chr01:7300791..7302992	Reverse	107	N	30			
GmCLE28a	Chr13:37349043..37349282	Reverse	83	N	27	80.3	PvCLE28	69.4
GmCLE28b	Chr12:38835186..38835383	Reverse	65	N	26			
GmCLE29a	Chr12:27615321..27615566	Forward	82	N	26	92.8	PvCLE29	84.3
GmCLE29b	Chr06:36330866..36331117	Reverse	83	N	26			
GmCLE30a	Chr06:36324860..36325095	Reverse	78	N	22	61.5	PvCLE30	60.5
GmCLE30b	Chr06:36255159..36255402	Reverse	81	N	26			
GmCLE31a	Chr07:37351348..37351668	Forward	106	N	22	92.5	–	–
GmCLE31b	Chr13:28570341..28570661	Reverse	106	N	22			
GmCLE32	Chr13:28559073..28559703	Reverse	68	N	23	–	–	–
GmCLE33a	Chr06:36402219..36402452	Reverse	78	N	23	84.4	PvCLE33	66.3
GmCLE33b	Chr12:27380684..27380911	Forward	76	N	24			
GmCLE34a	Chr12:38840660..38840902	Reverse	81	N	22	88.9	PvCLE34	78.6
GmCLE34b	Chr13:37353930..37354172	Reverse	81	N	22			
GmCLE35	Chr13:28564185..28564418	Reverse	78	N	23	–	PvCLE35	70.5
GmCLE36a	Chr13:34350525..34350935	Reverse	76	N	24	83.1	–	–
GmCLE36b	Chr15:6162182..6162415	Forward	77	N	25			
GmCLE37a	Chr16:4533525..4534140	Forward	185	Y	18	40.8	–	–
GmCLE37b	Chr19:35239153..35240209	Reverse	190	Y	24			
GmCLE40a	Chr12:3979297..3980162	Forward	82	Y	23	40.0	PvCLE40	47.9
GmCLE40b	Chr11:9961342..9961800	Forward	35	N	-			
GmCLV3a	Chr12:34902722..34903650	Forward	105	Y	28	93.3	PvCLV3	91.1
GmCLV3b	Chr13:40867356..40867942	Reverse	105	Y	29			
GmNIC1a	Chr12:36837550..36838464	Forward	80	N	22	86.3	PvNIC1	75.9
GmNIC1b	Chr13:39224711..39225630	Reverse	79	N	22			
GmRIC1a	Chr13:39215403..39216108	Reverse	95	N	28	77.3	PvRIC1	68.8
GmRIC1b	Chr12:36848528..36849475	Forward	96	N	27			
GmRIC2a	Chr06:47247215..47248215	Reverse	93	N	26	87.2	PvRIC2	74.5
GmRIC2b	Chr12:13187190..13187511	Forward	94	N	26			
GmTDIF1a	Chr07:41652868..41653137	Reverse	104	N	42	92.4	PvTDIF1	82.5
GmTDIF1b	Chr18:40563162..40564249	Reverse	104	N	41			
GmTDIF2a	Chr05:32724420..32724761	Reverse	113	N	28	92.2	PvTIDF2	87.9
GmTDIF2b	Chr08:6781787..6783296	Reverse	113	N	28			
GmTDIF3a	Chr09:4193781..4194815	Forward	125	N	31	76.7	PvTDIF3	68.6
GmTDIF3b	Chr15:13038523..13039541	Forward	127	N	29			

^*a*^ Number of amino acid residues.

^*b*^ After amino acid number listed.

Listed are the genetic location, pre-propeptide length, predicted intron presence, gene orientation, soybean and common bean homologue, pre-propeptide similarity (%). and SignalP signal peptide (SP) cleavage site.

**Table 2.** *Features of the common bean* (Phaseolus vulgaris*) CLE genes*

Name	Phytozome v10 ID	Pre-propeptide length^*a*^	Predicted intron	Chromosome location	Orientation	Oelkers *et al.* (2008)	uniprot.org
PvCLE1	Phvul.011G065200	96	Y	Chr11:5675757..5676469	Reverse	–	XP_007132079
PvCLE3	Phvul.006G092600	99	Y	Chr06:21113605..21114127	Forward	PvCLE169	XP_007147057
PvCLE4	Phvul.002G008500	67	N	Chr02:960456..961284	Reverse	–	XP_007156683
PvCLE5	Phvul.003G035700	121	N	Chr03:3588969..3589711	Forward	–	XP_007153443
PvCLE6	Phvul.007G027300	94	Y	Chr07:2049797..2054614	Reverse	PvCLE176	XP_007142910
PvCLE7	Phvul.002G085300	108	N	Chr02:13297480..13297806	Forward	–	XP_007157625
PvCLE8	Phvul.009G187200	95	N	Chr09:27684592..27685489	Forward	–	XP_007138182
PvCLE9	Phvul.003G190100	95	N	Chr03:40210422..40210709	Forward	–	XP_007155310
PvCLE10	Phvul.002G079000	101	Y	Chr02:11819569..11820862	Reverse	–	XP_007157554
PvCLE11	Phvul.001G025500	77	N	Chr01:2309373..2309606	Reverse	–	XP_007160889
PvCLE12	Phvul.004G023800	108	Y	Chr04:2459046..2460734	Reverse	–	XP_007151170
PvCLE13	Phvul.005G069900	102	Y	Chr05:11484552..11485119	Reverse	–	XP_007149431
PvCLE14	Phvul.007G068800	88	N	Chr07:6196473..6196739	Reverse	–	XP_007143392
PvCLE15a	Phvul.007G068400	85	N	Chr07:6165176..6165433	Reverse	–	XP_007143388
PvCLE15b	Phvul.007G068500	83	N	Chr07:6181155..6181406	Forward	–	XP_007143389
PvCLE15c	Phvul.007G068600	87	N	Chr07:6184216..6184479	Reverse	–	XP_007143390
PvCLE15d	Phvul.007G068700	84	N	Chr07:6189914..6190168	Forward	–	XP_007143391
PvCLE16	Phvul.004G117600	86	N	Chr04:38385127..38385862	Forward	–	XP_007152295
PvCLE17	Phvul.002G287300	97	N	Chr02:45090923..45091742	Reverse	–	XP_007160038
PvCLE18	Phvul.003G137800	85	N	Chr03:33013056..33013313	Reverse	–	XP_007154669
PvCLE19	Phvul.002G095900	104	Y	Chr02:17549689..17550064	Forward	–	XP_007157755
PvCLE20	Phvul.001G120900	92	N	Chr01:34104465..34105721	Forward	–	XP_007162068
PvCLE21	Phvul.008G203000	88	N	Chr08:51319273..51319539	Forward	–	XP_007141519
PvCLE22	Phvul.006G016000	90	N	Chr06:7671543..7672241	Reverse	-	XP_007146145
PvCLE23	Phvul.008G211300	74	N	Chr08:52313956..52316136	Forward	–	XP_007141620
PvCLE24	Phvul.007G101800	109	N	Chr07:11339237..11339566	Reverse	–	XP_007143789
PvCLE25	Phvul.003G177600	110	N	Chr03:38979082..38979719	Forward	–	XP_007155150
PvCLE26	Phvul.002G168200	85	Y	Chr02:31082684..31084138	Reverse	–	XP_007158622
PvCLE27	Phvul.002G081400	106	N	Chr02:12270950..12272253	Reverse	–	XP_007157583
PvCLE28	Phvul.005G067900	83	N	Chr05:10636536..10636787	Reverse	–	XP_007149409
PvCLE29	Phvul.011G160600	81	N	Chr11:42316953..42317385	Forward	–	XP_007133207
PvCLE30	Phvul.011G160700	82	N	Chr11:42325813..42326352	Forward	–	XP_007133208
PvCLE31	Chr01: 14906066..14906353	95	N	Chr01: 14906066..14906353	Forward	–	–
PvCLE33	Chr11:42291102..42291350	82	N	Chr11:42291102..42291350	Reverse	-	-
PvCLE34	Chr05:10644869..10645097	75	N	Chr05:10644869..10645097	Reverse	–	–
PvCLE35	Phvul.003G057900	75	N	Chr03:7610340..7610764	Forward	–	XP_007153705
PvCLE40	Phvul.011G056800	114	Y	Chr11:4877577..4878010	Forward	–	XP_007131981
PvCLV3	Phvul.005G120600	104	Y	Chr05:34343926..34344486	Reverse	–	XP_007150035
PvNIC1	Phvul.005G097000	80	N	Chr05:28793851..28794118	Reverse	–	XP_007149764
PvRIC1	Phvul.005G096900	115	Y	Chr05:28775368..28775758	Reverse	–	–
PvRIC2	Phvul.011G135900	93	N	Chr11:30985821..30986626	Reverse	–	XP_007132915
PvTDIF1	Phvul.008G124100	118	N	Chr08:17187233..17187933	Forward	–	XP_007140575
PvTDIF2	Phvul.002G187400	108	N	Chr02:34265616..34266385	Forward	–	XP_007158853
PvTDIF3	Phvul.009G244400	115	N	Chr09:35772334..35773004	Reverse	–	XP_007138869

^*a*^ Number of amino acid residues.

Listed are the genetic location, pre-propeptide length, and predicted intron presence.

The identified genes are scattered across the genomes, with at least one located on every chromosome, except for chromosome 10 of common bean. Chromosome 13 of soybean contains the most CLE peptide-encoding genes, with a total of 12. Most of the identified genes lack predicted introns, with the exception of 12 soybean genes and nine common bean genes (Tables 1, 2).

Many of the genes identified here had not been discovered previously and therefore had not yet been assigned a name. In contrast, those which were previously reported had as many as five different aliases. To unify the nomenclature, designations were assigned based on the names of all previously characterized soybean CLE peptides (e.g. Cock and McCormick, 2001; Reid *et al.*, 2011*a*; Wong *et al.*, 2013), and the *Arabidopsis* phylogenetic approach was used for all non-characterized genes (Cock and McCormick, 2001). The duplicated nature of the soybean genome was also accounted for by identifying *a* and *b* copies of homeologous gene pairs (described below). In common bean, the gene names were assigned based on their orthologue in soybean (Table 1; Supplementary Fig. S1 available at *JXB* online). A comprehensive list of all soybean and common bean names, including all previous identifiers, is provided in Supplementary Table S1.

Aside from plants, cyst nematodes are the only known organisms to possess CLE peptide-encoding genes (Mitchum *et al.*, 2013). These peptides appear to assist in parasitism of the host. To determine whether mutualistic symbiotic organisms also encode for CLE peptides that assist in infection, a protein search of mycorrhiza (http://genome.jgi.doe.gov/) and rhizobia (Rhizobase; http://genome.microbedb.jp/rhizobase; Fujisawa *et al.* 2014) species was conducted using CLE domain consensus sequences and also pre-propeptide sequences. This thorough search yielded the identification of no CLE peptide-encoding genes in these organisms.

### Identification of homeologues and orthologues in soybean and common bean

To characterize their amino acid sequences, all identified CLE peptide-encoding genes were translated and successive multiple sequence alignments were conducted using entire CLE pre-propeptide sequences. Despite having large variable domains, the pre-propeptides grouped strongly according to their CLE domain sequence in both soybean (Fig. 1) and common bean (Fig. 2). This helped in identifying likely homeologous (duplicate) copies of genes in the palaeopolyploid genome of soybean, with 39 pairs identified compared with only six genes having no duplicate (Fig. 1; Table 1). The six genes lacking a duplicate were re-blasted against the soybean genome to confirm their lack of a duplicate, and their homeologous chromosome region was checked for unannotated genes. The presence of a common bean orthologue confirmed they were not triplicated within the soybean genome.

To identify likely orthologues between soybean and common bean, an additional multiple sequence alignment was produced using the CLE peptide-encoding gene families of both species (data not shown). This alignment was also useful in confirming the 39 homeologous gene pairs of soybean. As expected, all previously reported gene orthologues of soybean and common bean clustered together (e.g. RIC, NIC; Ferguson *et al.*, 2014). Additional orthologue candidates also clustered; however, soybean has four homeologous gene pairs and one individual gene lacking an apparent duplicate that appear to have no orthologue in common bean (*GmCLE2a* and *b*; *GmCLE31a* and *b*; *GmCLE32*; *GmCLE36a* and *b*; and *GmCLE37a* and *b*; Table 1).

**Fig. 3.**
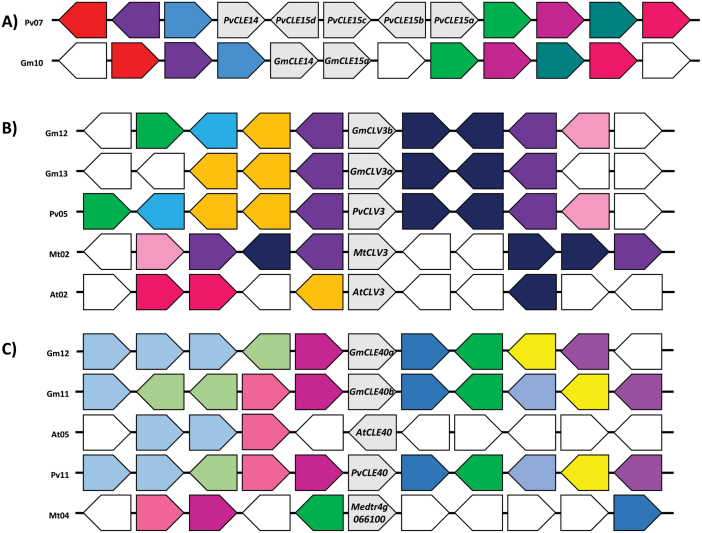
Genomic environment of *PvCLE15* tandemly duplicate genes of common bean, and the *CLV3* and *CLE40* genes of different species. The genes of interest are positioned centrally and shaded in grey. Species and chromosome number are indicated to the left of each genomic segment. Surrounding genes similar in putative function are indicated by the same colour and genes with unrelated putative functions are uncolored. The direction of the arrow represents the orientation of the gene compared with that of the CLE gene. (A) Common bean chromosome 7 containing a tandem gene duplication not found on the orthologous region of soybean on chromosome 10. Orthologues of (B) *CLV3* and (C) *CLE40* in soybean, common bean, *Arabidopsis*, and *M. truncatula*. A high level of genetic synteny is shown here for each of these CLE genes.

When identifying gene orthologues, it was noticed that three of the 44 genes identified in common bean did not have an apparent orthologue in soybean (Table 1; Supplementary Fig. S1 at *JXB* online). These genes are all part of a group of four tandemly duplicated genes located on chromosome 7, called *PvCLE15a*, *b*, *c*, and *d*, and thus can all be considered orthologous to the same genes in soybean, *GmCLE15a* and *b*. This indicates that the tandem duplication occurred in common bean after it diverged ~19 MYA from soybean. Directly upstream of these tandemly duplicated genes and adjacent to *PvCLE15d* is another CLE peptide-encoding gene, *PvCLE14* (Fig. 3A). This tandem duplication also occurs in soybean (*GmCLE14* and *GmCLE15a*) and thus must have occurred prior to the two species diverging.

Two additional sets of genes occur in tandem in common bean: *PvCLE29* and *PvCLE30*, and *PvNIC1* and *PvRIC1*. In soybean, the *NIC1* and *RIC1* genes also occur in tandem, suggesting that this duplication occurred prior to the divergence of soybean and common bean. However, due to the whole-genome duplication, soybean has homeologous regions that include these genes, resulting in two tandem repeats: *GmNIC1a* and *GmRIC1b* on chromosome 12 and *GmNIC1b* and *GmRIC1a* on chromosome 13.

Manual adjustments were made to some coding sequences predicted in Phytozome regarding the placement of their start codon. These adjustments were based on sequence similarity to their duplicate gene, to clustering sequences in common bean (i.e. probable orthologues), and/or to signal peptide domain prediction results (described below). In total, eight soybean sequences were trimmed slightly to place their start codon downstream of where it was predicted in Phytozome (GmCLE10b, GmCLE16b, GmCLE21b, GmCLV3b, GmTDIF1a, GmTDIF1b, GmRIC1a, and GmRIC2b). An additional five sequences were extended to include a start codon slightly upstream of that predicted in Phytozome (GmCLE3a, GmCLE16a, GmCLE20a, GmCLE27a, and GmCLE28a).

### Characterization of CLE pre-propeptides in soybean and common bean

CLE pre-propeptides typically consist of a signal peptide, a variable domain, and a CLE domain, with some also having a C-terminal extension (Hastwell *et al.*, 2015). All of the CLE pre-propeptides identified here have this structure. Moreover, they are rich in lysine (11.4%) and serine (11.3%), and are notably poor in cysteine (1.3%), tyrosine (1.3%), and tryptophan (0.7%; often poorly represented in plants) (Supplementary Table S2 at *JXB* online), which is typical amongst CLE peptides (Hastwell *et al.*, 2015). The length of the CLE pre-propeptides varies, with the smallest being 67 residues in both soybean and common bean (excluding likely pseudogenes reported below), and the longest being 127 and 121 residues, respectively. Some contain histidine repeats in their variable domain, but this does not correlate with sequence length.

The signal peptide located at the N-terminus of the pre-propeptide is typically hydrophobic and is responsible for exporting the propeptide from the cell (Rojo *et al.*, 2002). Hydrophobicity analysis confirmed that the signal peptide is the most hydrophobic region of the CLE pre-propeptides investigated here, whereas the remaining propeptide is more hydrophilic, as determined by Kyte and Doolittle (1982) scores (Supplementary Fig. S2 at *JXB* online). Indeed, 61.4% of the amino acid residues occurring in the signal peptide domain are hydrophobic (Supplementary Fig. S2). SignalP prediction software was used to determine the putative cleavage site of the signal peptide (Table 1). Using these predicted signal peptide sequences, a multiple sequence alignment and phylogenetic tree was constructed that showed less conserved and confident groupings (data not shown) compared with entire pre-propeptides. One pre-propeptide, GmCLE40b, is not predicted to have a signal peptide, as it is truncated and only 34 amino acids in length (Table 1; Fig. 1).

Directly following the signal peptide domain in the pre-propeptide is the variable domain. This region only shows conservation between homeologous and/or orthologous genes (Figs 1, 2). However, the final residue of the variable domain positioned directly before the CLE domain is commonly a lysine (48.4%), with asparagine (13.9%), glutamic acid (9.0%), alanine (7.4%), and histidine (5.7%) as the next four highest represented amino acids at this position.

The CLE domain represents the region of the pre-propeptide that is cleaved and modified to become the functional CLE peptide product. Of the 126 CLE peptide-encoding genes of soybean and common bean, there are 54 unique CLE domain sequences that are 12 amino acids in length (with 44 of 82 in soybean and 40 of 44 in common bean). This number increases to 60 sequences if 13 amino acids are taken into account. All mature CLE peptides that have been biochemically confirmed to date have been 13 amino acids in length (Ohyama *et al.*, 2009; Shinohara *et al.*, 2012; Okamoto *et al.*, 2013; Chen *et al.*, 2015); however, only 54.8% of the pre-propeptide CLE sequences of soybean and common bean have a residue in position 13, with the others having a stop codon preventing them from being any more than 12 amino acids in length.

Sequence similarity within the CLE pre-propeptides of soybean and common bean is highest in the CLE domain (Figs 1, 2). There is no 100% conserved residue, although position 12 has a highly conservative histidine/asparagine substitution. The least conserved residues are at position 2 (15.8% pairwise identity) and position 5 (19.7% pairwise identity). Of the critical residues previously identified in the CLE domain (e.g. Ni *et al.*, 2011; Reid *et al.*, 2013), position 1 is predominantly arginine, or, in some cases, histidine (i.e. TDIF peptides). An additional group has threonine at position 1 (GmCLE16a, GmCLE16b, and PvCLE16). Three others that group together have valine, lysine, and leucine residues at this position (PvCLE15a, PvCLE15d, and GmCLE15b, respectively; Figs 1, 2), which includes two of the four common bean genes that are tandemly duplicated (described above). Position 7, which is often post-translationally modified, is predominately a proline. However, there are 10 soybean homeologues and five associated common bean orthologues where a serine (CLE7; CLE8; CLE11 and CLE23 orthologous) or alanine (CLE4 orthologues) is in that position. Interestingly, soybean has six pairs (i.e. 12 genes) of homeologous CLE peptide-encoding genes that have a mismatch within their CLE domain as a result of naturally occurring mutations (Fig. 1). The impact of amino acid changes on the function and activity of various *Arabidopsis* and legume CLE pre-propeptides was recently reviewed (Hastwell *et al.*, 2015).

Some CLE pre-propeptides contain a fourth domain directly following the CLE domain, called the C-terminal extension. The precise function of this domain remains unclear. Only 32.5% of the CLE pre-propeptides in soybean and common bean have this domain, similar to the CLE pre-propeptide family of *A. thaliana* (31.3%; Cock and McCormick, 2001). The only prevalent feature of the C-terminal extension appears to be the common presence of proline (19.5%). Indeed, the sequence is highly variable in length and amino acid residues, except between homeologous and/or orthologous genes (Fig. 1). Interestingly, the domain is present in 83.3% of the CLE genes that contain a predicted intron. It is also present in CLV3 orthologues and in almost all rhizobia-induced nodulation-suppressing CLE peptides (with the exception of MtCLE12; Hastwell *et al.*, 2015).

### Pseudogenes and multi-CLE peptide-encoding genes of soybean and common bean

Due to insertion, duplication, and deletion events, some of the CLE peptide-encoding genes identified here do not fit the common tripartite domain structure. For example, in soybean, *GmCLE28b*, *GmCLE30b*, and *GmCLE40b* are all probably pseudogenes. *GmCLE28b* and *GmCLE40b* have nonsense mutations that result in a truncation prior to the CLE domain. However, the sequences downstream of these mutations align closely to *GmCLE28a* and *GmCLE40a*, respectively. *GmCLE30b* has low conservation in the CLE domain after residue five, when compared with its duplicate, *GmCLE30a*. This appears to be due to a deletion event causing a frameshift directly in the CLE domain. It is likely that none of these three pseudogenes genes produces a functional CLE peptide. They have been denoted as the *b* copy, consistent with the *RIC*, *NIC*, and *CLV3* genes, where the *b* copy may not be transcribed/functional (Reid *et al.*, 2011*a*; Wong *et al.*, 2013).

**Fig. 4.**
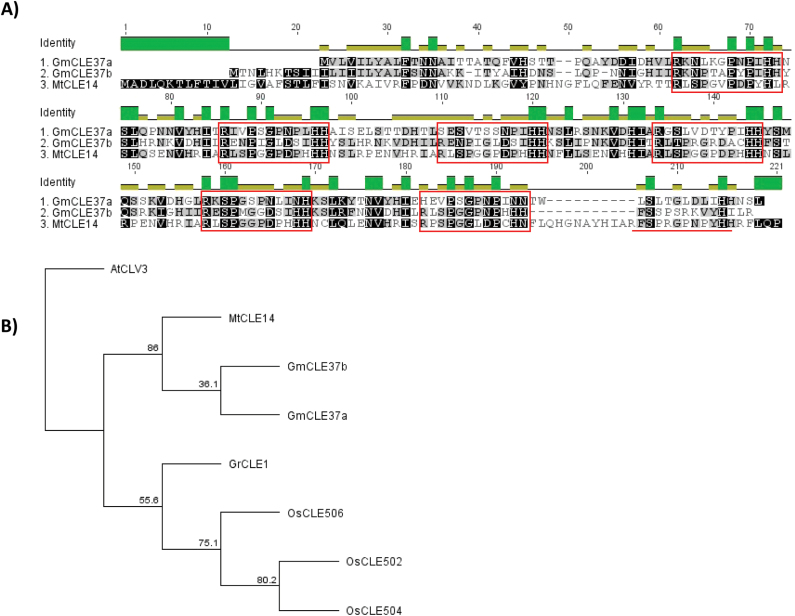
Multi-CLE domain pre-propeptides. (A) Multiple sequence alignment of the soybean and *M. truncatula* multi-CLE domain pre-propeptides, with putative 13 amino acid residue CLE domains highlighted by a red box. An additional CLE domain of MtCLE14 that is not detected in the two soybean pre-propeptides is underlined in red. Four MtCLE14 CLE domains are identical in sequence (CLE domains 2–5) while there are no 100% conserved 13 amino acid residue CLE domains in soybean. However, there are two fully conserved 12 residue CLE domains in GmCLE37b (CLE domains 1 and 2). (B) Phylogenetic tree of known multi-CLE domain-containing pre-propeptides of rice (*Oryza sativa*), potato cyst nematode (*Globodera rostochiensis*), *MtCLE14* of *M. truncatula*, and the newly identified *GmCLE27a* and *GmCLE37b* of soybean, including AtCLV3 as an outgroup. The multi-CLE domain pre-propeptides identified here cluster separately from those that were previously identified. The tree is shown with bootstrap confidence values expressed as a percentage from 1000 bootstrap replications.

Genes encoding pre-propeptides that contain multi-CLE domains were also identified. This includes *GmCLE37a* and *GmCLE37b*, which have six possible CLE domains each (Fig. 4A). These were excluded from the alignment in Fig. 1 as they do not have the archetypical domain structure. There are only two identical CLE domains within the soybean multi-CLE domain pre-propeptides and they both occur in GmCLE37b (Fig. 4A). A multi-CLE domain-containing pre-propeptide previously reported in *Medicago truncatula* by Oelkers *et al.* (2008) was identified here as *MtCLV3* (*MtCLV3* was previously discovered by Chen *et al.*, 2009, but was not reported to encode a multi-CLE domain). Although *MtCLV3* encodes three CLE domains, only one is actually translated due to the presence of a previously undetected intron identified here. An additional pre-propeptide of *M. truncatula*, called MtCLE14, contains a multi-CLE domain with seven CLE peptide domains (Fig 4A; Mortier *et al.*, 2011). MtCLE14 contains four identical 12 amino acid CLE domains in tandem, each followed by an asparagine residue (possible representing a 13th residue in the CLE peptide), and each preceded by the same two hydrophobic residues (Fig. 4A).

In *A. thaliana*, *AtCLE18* encodes both a CLE and a CLEL domain (Meng *et al.*, 2012). TBLASTN and BLASTN searches of the soybean and common bean genomes failed to identify a similar gene. Multi-CLE domain-encoding genes of nematodes are processed into single functional CLE peptide ligands (Chen *et al.*, 2015). TBLASTN searches of the soybean and common bean genomes using the known multi-CLE domain-encoding gene of nematode and three others of rice (Olsen and Skriver, 2003; Oelkers *et al.*, 2008) identified no orthologues. A phylogenetic analysis (Fig. 4B) also shows that the legume multi-CLE domain pre-propeptides cluster separately from the nematode and rice pre-propeptides.

### Categorization and functional predictions of soybean CLE peptides

**Fig. 5.**
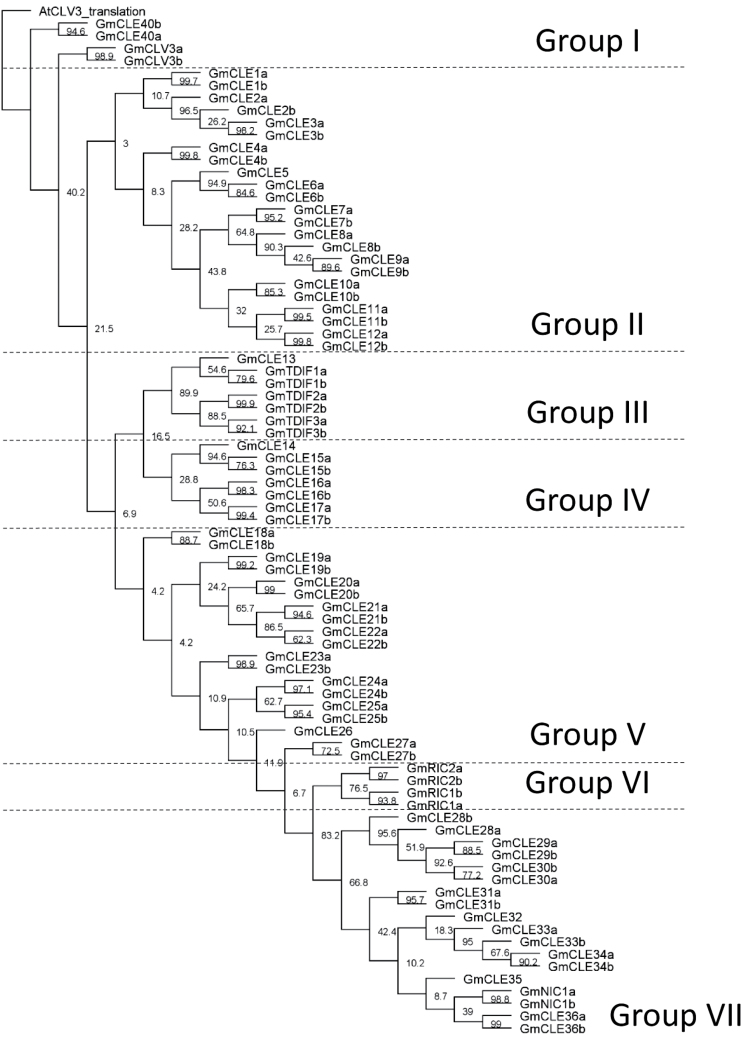
Soybean CLE pre-propeptide phylogenetic tree illustrating the seven distinct identity groups. Phylogenetic analysis was performed using the multiple sequence alignment generated with entire pre-propeptide sequences (Fig. 1), including AtCLV3 as an outgroup. Homeologous genes consistently cluster together with high confidence (indicated by high bootstrap values). The seven groups (Group I–VII) were assigned based on clustering in the tree, in addition to sequence similarity. The tree is shown with bootstrap confidence values expressed as a percentage from 1000 bootstrap replications.

**Fig. 6.**
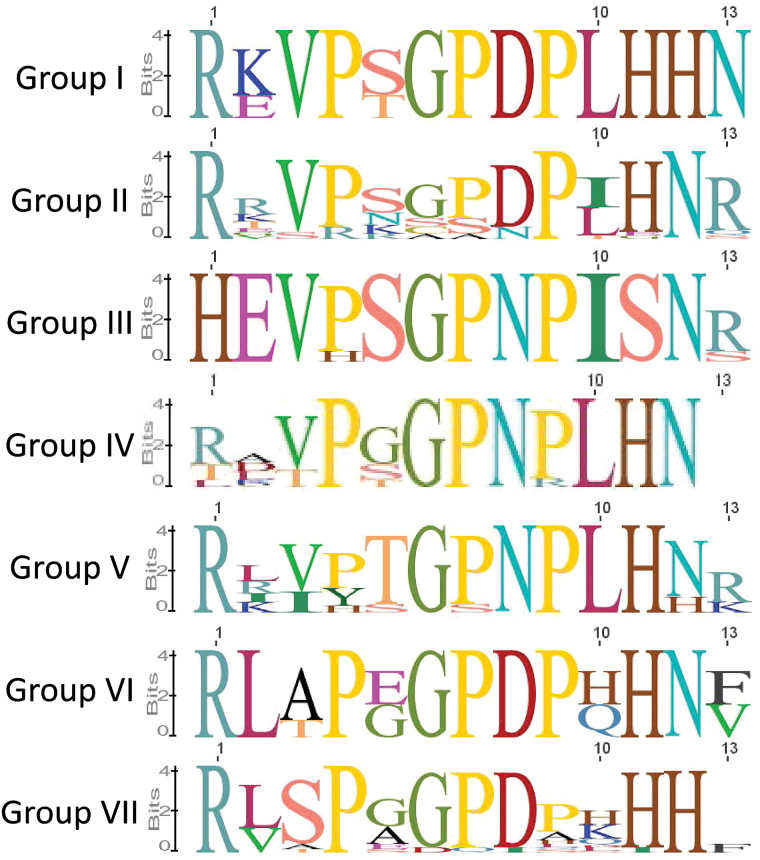
CLE domain consensus sequences from the seven soybean pre-propeptide groups. Logo diagrams illustrate the 13 amino acid CLE domain consensus sequences for soybean CLE Groups I–VII, as determined from multiple sequence alignments generated for each group. The 13th amino acid is a consensus of only those sequences that have a residue at that position. Group IV does not have any residues at that position and hence the logo diagram for this group is 12 residues only.

The function of many CLE peptides can be predicted based on sequence. The *Arabidopsis* CLE peptides are currently categorized into two groups: type-A affecting root and shoot meristem development, and type-B affecting vasculature development (Matsubayashi, 2014). The soybean CLE peptides were assigned into different categories based on the sequence alignment, phylogenetic grouping of their pre-propeptides, and their functional roles where known. The groups were initially defined based on phylogenetic analysis, and were then further refined following examination of their CLE domain and adjacent residues. In total, seven groups (Groups I–VII) were identified (Fig. 5). Logo alignments (Fig. 6) were subsequently constructed to establish the level of conservation within the 13 amino acid CLE domain of each group, with highly conserved residues probably critical to their function.

Group I is small, consisting of only four members. It contains CLV3, CLE40, and their homeologous duplicates (Fig. 5). CLV3 and CLE40 are well characterized and are responsible for apical meristem regulation in the shoot and root, respectively (Grienenberger and Fletcher, 2015). The CLE domain of this group is highly conserved (Fig. 6), particularly for amino acid residues reported to be critical for function (Song *et al.*, 2013).

Group II contains the least conserved CLE domain of all the established groups. It is also the largest group, with 23 members, which may account for it having the lowest degree of conservation (Figs 5, 6). The group cannot be divided further with any degree of confidence using a phylogenetic approach. Interestingly, it has low conservation at residue six, which is generally considered to be critical for function, possibly having a role in enabling the CLE peptide to rotate or bend (Hastwell *et al.*, 2015). Most of the CLE peptides in this group remain poorly characterized in any species; however, some of the soybean CLE pre-propeptides show similarity to, and group closely with, AtCLE45 (Supplementary Fig. S3 at *JXB* online).

Group III contains seven members, including the three TDIF pre-propeptides and their homeologues, in addition to one other member of unknown function that lacks a duplicate copy (Fig. 5). This group is orthologous to the *Arabidopsis* type-B CLE pre-propeptides that influence vasculature development, including AtCLE41, ACLE42, and AtCLE44 (Fig. 5; Supplementary Fig. S3 at *JXB* online; Matsubayashi, 2014). A defining feature of this soybean group is that all of the CLE peptides begin with a histidine residue, as opposed to the classical arginine (Fig. 6). Interestingly, with the exception of the non-TDIF peptide (GmCLE13), the 12 amino acid CLE domain is 100% conserved. Also of note is that the members of this group are the only CLE peptides to have a serine residue at position 11, rather than the characteristic histidine (Fig. 6).

Group IV consists of seven members and notably does not encode any CLE peptides that are 13 amino acids in length (Fig. 6). It is also the group that is least conserved at residue one. The function of the group members remains poorly defined.

Group V is another large group, having 19 members (Fig. 5). Of the CLE peptides encoded by this group, all but one contain an acidic amino acid (glutamic acid or aspartic acid) and a lysine residue immediately preceding the CLE domain (Fig. 1). The CLE peptides encoded by this group also predominantly have a threonine at position 5, which is not characteristic of any of the other groups (Fig. 6).

Group VI is a small group consisting entirely of the rhizobia-induced CLE peptides (RICs) and their homeologous copies (Fig. 5). This group has been well characterized for their role in regulating legume nodule development (reviewed in Hastwell *et al.*, 2015), including the identification of amino acid residues in the CLE domain that are critical for function (Reid *et al.*, 2013).

Group VII consists of 18 members, and, like Group I, has two histidine residues located at positions 11 and 12 (Figs 5, 6). It contains the majority of the genes that were unpredicted in Phytozome (Table 1). The function of most remains unknown; however, it does include the nitrate-induced CLE peptide (NIC1a) and its homeologue, NIC1b (Reid *et al.*, 2011*a*; referred to as NIC2 in Lim *et al.*, 2014), that is well known for its role in controlling legume nodulation in response to the nitrogenous content of the rhizosphere (reviewed in Hastwell *et al.*, 2015).

These groupings hold true when the common bean CLE pre-propeptides are added to the phylogenetic analysis with soybean (Supplementary Fig. S1 at *JXB* online). When *Arabidopsis* is also included (Supplementary Fig, S3), the groupings are still conserved generally, but are supported by lower bootstrap proportions, especially Group II. This is not surprising when dealing with >150 pre-propeptides from three different species and, even though some groups are divided further when a non-legume is included, the larger groups cannot be confidently split further based on the low bootstrap proportions. In all instances, Group III is supported by very high bootstrap proportions (>88).

A C-terminal extension is encoded by one-third of the genes identified here, spanning across the various groups, but predominantly being found in Groups I, II, and VI (Figs 1, 5). GmCLE31a and b, and GmCLE13, also contain a C-terminal extension. The presence of a predicted intron correlates slightly with the groupings, as all of the genes in Group I contain a predicted intron, as do some in Group II, but none in Groups III–VII, with the exception of *GmCLE13* (Group III), which incidentally also contains the only CLE domain sequence divergence of its group, as noted above (Table 2; Figs 1, 5, 6).

**Fig. 7.**
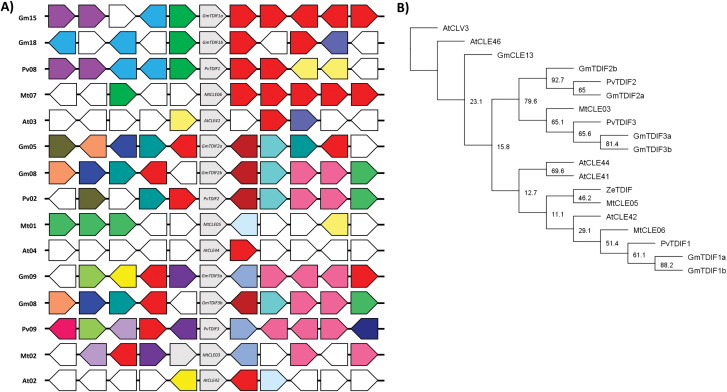
TDIF genes in soybean, common bean, *Arabidopsis*, *Zinnia elegans*, and *M. truncatula*. (A) Genomic environments of the TDIF-encoding genes highlight the genetic synteny between the genes identified here in soybean, common bean, and *M. truncatula* with previously characterized TDIF genes of *A. thaliana*, *AtCLE41*, *AtCLE42*, and *AtCLE44*. TDIF-encoding genes are shown positioned centrally and shaded in grey. Species and chromosome number are indicated to the left of each genomic segment. Surrounding genes similar in putative function are indicated by the same colour and genes with unrelated putative functions are uncoloured. The direction of the arrow represents the orientation of the gene compared with that of the CLE gene. A high level of genetic synteny is shown here for each of the predicted TDIF-encoding genes, but was not found for *AtCLE46* and *GmCLE13* (data not shown), whose CLE domain begins with a histidine residue but is not a TDIF peptide. (B) Phylogenetic tree of TDIF-encoding pre-propeptides, including ZeTDIF, and also AtCLV3 as an outgroup. Two pre-propeptides, AtCLE46 and GmCLE13, are also included that have CLE domains beginning with a histidine residue, but are not true TDIF CLE peptides and did not group with the TDIF pre-propeptides. The tree is shown with bootstrap confidence values expressed as a percentage from 1000 bootstrap replications.

The groupings described here could help in elucidating the function of CLE peptides where a function is yet to be assigned. Indeed, these groupings, together with genomic environment analyses, were used to identify previously unknown soybean and/or common bean orthologues of AtCLV3-, AtCLE40-, and TDIF-encoding genes, as well as likely *M. truncatula* orthologues. *AtCLV3* was the first CLE gene to be identified in any species (Fletcher *et al.*, 1999) and has since been identified in soybean and *M. truncatula* (*GmCLV3a*, *GmCLV3b*, and *MtCLV3*; Chen *et al.*, 2009; Wong *et al.*, 2013). Investigations into the genomic environment and pre-propeptide sequence similarity (Fig. 3B) led to the identification of a CLV3 orthologue in common bean. Similar approaches were used to identify *AtCLE40* orthologues (Fig. 3C) in common bean and *M. truncatula*, in addition to *GmCLE40b*, the homeologue of *GmCLE40a*. Moreover, all TDIF orthologues in soybean, common bean, and *M. truncatula* were established (Fig. 7). In contrast, despite *AtCLE46* and *GmCLE13* sharing a high level of sequence similarity in the CLE domain, they do not show synteny to the TDIF genes, or to each other, and cluster separately (Fig. 7). Thus, these genes are unlikely to be true TDIF peptides.

### Expression analysis of CLE peptide-encoding genes of soybean, common bean, and *Arabidopsis*

A meta-analysis of the publicly available transcriptome data was conducted in soybean, common bean, and *Arabidopsis* (Supplementary Tables S3–S5 at *JXB* online). The transcriptomic expression of functionally characterized soybean and common bean CLE peptide-encoding genes was consistent with the literature (i.e. RICs and NIC1, Reid *et al.*, 2011*a*; Ferguson *et al.*, 2014). Interestingly, there were no transcriptional data available for CLV3 orthologues in soybean and common bean (Supplementary Tables S3, S4).

Trends observed in the expression of CLE peptide-encoding gene orthologues across different tissues of soybean and common bean were also consistent (Supplementary Tables S3, S4 at *JXB* online). For example: *PvCLE10*, *GmCLE10a*, and *GmCLE10b* showed varying levels of expression across all tissue types, in a similar trend; *PvCLE17* and *GmCLE17a* are expressed in all tissue types except seeds, flowers, and early pod growth; and *PvCLE19* and *GmCLE19a* show expression in all tissues except mature nodules. These three orthologous gene groups (CLE10, CLE17, and CLE19) also show high (>93) bootstrap values in the phylogenetic analyses (Supplementary Fig. S2). In contrast, *CLE24* showed different expression patterns between soybean and common bean orthologues. *GmCLE21a* and *GmCLE21b* show the same expression trends, but *PvCLE21* transcripts were only detected in the early seed development stage. In soybean, where data were available for both the *a* and *b* copy, the general trend of expression was consistent but in most cases the level or the time of expression varied. There is no consistent expression pattern between pre-propeptides belonging to soybean Groups I–VII, but closely related peptides probably perform a similar role in different developmental tissues as with the TDIF orthologues (Supplementary Tables S3–S5; Matsubayahsi, 2014).

To determine if expression trends are similar between orthologues of soybean, common bean, and *Arabidopsis*, and to see how orthologues clusters, a phylogenetic tree of the pre-propeptides from the three species was produced (Supplementary Fig. S3 at *JXB* online). Branches that were supported by >50 bootstrap proportions include AtCLE46 and CLE1; AtCLE21 and CLE4; AtCLE27 and CLE6; AtCLE20 and CLE23; AtCLE12 and CLE24; and the cluster containing the TDIF orthologous genes, as established previously in Fig. 7.

As expected, the legume orthologues show a similar expression trend for each of these branches and, in the case of AtCLE12, a similar trend was observed with GmCLE24a and PvCLE24 (Supplementary Tables S3–S5 at *JXB* online). Interestingly, AtCLE27 and AtCLE21 were not expressed in any tissues, similar to the case of their respective and related legume pre-propeptides (Supplementary Fig. S3). All the TDIF orthologues with available expression profiles show a highly similar pattern (Supplementary Tables S3–S5).

Within the meta-analysis of the transcriptomes, interesting candidates were identified as targets for future functional characterization. PvCLE29 was found only in the flower at a very high level; PvCLE24 shows very high root and nodule expression (Supplementary Table S4 at *JXB* online); and GmCLE25a is only expressed in root tissue (Supplementary Table S3).

The meta-analysis shows similar trends for orthologous genes. However, to date, only one-third of the CLE peptide-encoding genes of soybean, and less than half from *Arabidopsis*, are represented. It is also likely that some genes that respond to external stimuli (e.g. rhizobia for *RIC1* and *2* and nitrate for the *NIC1* orthologues) were not induced if the required treatment was not part of the study.

Feeding studies were not attempted here because the precise size and modification of each of the novel peptides is completely unknown. Although feeding unmodified or semi-modified synthetic peptides could be attempted, the peptides being fed would be designed based on prediction (in terms of both length and modifications). Furthermore, they would be applied in unnaturally high concentrations, without regard to temporal or spatial regulation, to a broad range of tissues and cell types to which they might not normally localize. These issues would be further exacerbated in feeding studies using roots grown on agar containing high levels of sucrose and nitrate, and exposed to light. Such studies would result in an extremely high frequency of false-positive outcomes that are of little biological value. For comparison sake, an ecologist investigating the impact of wild boars on the environment would not flood a forest with hams. Indeed, it has readily been shown that CLE peptides altered from their correct modification, size, and location can induce a phenotypic effect in feeding (e.g. Fiers *et al.*, 2005; Whitford *et al.*, 2008; Ohyama *et al.*, 2009; Mortier *et al.*, 2010; Kondo *et al.*, 2011) or site-directed mutagenesis and domain-swap studies (e.g. Ni and Clark, 2006; Song *et al*., 2012; Reid *et al.*, 2013). CLE peptides unlikely to come into contact with a given receptor can be forced to bind to that receptor *in vitro* (as elegantly demonstrated by Shinohara and Matsubayashi, 2015). Thus, results from peptide feeding studies may not be biologically relevant, and any phenotypic changes observed would need to be interpreted with extreme caution. For these reasons, the focus here was to use alternative approaches to help determine the role of novel peptides of unknown structure and function.

## Discussion

CLE peptides are widely recognized as important contributors to plant signalling and development; however, a lot remains to be understood about these critical signal molecules. Here, this emerging field was enhanced by the discovery and categorization of the CLE peptide families of soybean and common bean, two of the world’s most agriculturally important crops. A total of 84 CLE peptide-encoding genes in soybean and 44 in common bean were identified, and subsequently an array of bioinformatic approaches were conducted for comparative genomic and molecular evolution analyses. Doing so led to the identification of three pseudogenes, two multi-CLE domain-encoding genes in soybean, and a tandem gene duplication event in common bean. It also enabled the establishment of all homeologous gene copies within soybean, and orthologous copies amongst soybean, common bean, and *Arabidopsis*. Searches using rhizobia and mycorrhiza genomes were also performed, but revealed no CLE peptide-encoding genes in these organisms. Thus, to date, CLE peptides appear to be exclusive to plants and nematodes.

The function of most CLE peptides remains completely unknown. However, phylogenetic analyses of the entire CLE pre-propeptide families of soybean, common bean, and *Arabidopsis* show that they group strongly according to their CLE domain and known/predicted function. Based on the analyses, it is demonstrated that the soybean CLE pre-propeptides (excluding multi-CLE domain-encoding genes) grouped into seven distinct categories (Groups I–VII) and that these groups are generally preserved when other species are included. This expands on the two groups reported in *Arabidopsis* (type-A affecting root and shoot development, and type-B affecting vasculature development; e.g. Matsubayashi, 2014). The categorization approach reported here could be a useful tool for elucidating the function of unknown CLE peptides and their closely related homeologous and orthologous sequences. As an example, all known CLE peptides of similar function were found to group together (CLV3 and CLV40 formed Group I, the TDIFs formed Group III, and the RICs formed Group VI). Moreover, the groupings revealed a number of highly conserved amino acid residues present in the peptide domains of each group, which are probably central to the activity of their ligands.

The groups identified here include peptides performing a similar developmental role in a range of different tissues, as exemplified by Group III, whose *Arabidopsis* orthologues are known to have the same function (Matsubayashi, 2014) but are expressed in a range of different tissues. This is also seen with the Group I and Group VI peptides. Given that the genes encoding the members of these groups do not show consistent expression patterns, it is possible that they too may have similar roles in different tissues. Furthermore, the transcriptome evidence presented here provides some insight into where the peptides function, as they often act in a local manner (Matsubayashi, 2014). Indeed, the only known CLE peptides to act systemically are those involved in the autoregulation of nodulation signalling pathway of legumes (Hastwell *et al.*, 2015).

The ancestral genome shared by soybean and common bean duplicated ~59 MYA and subsequently reconverged (Schmutz *et al.*, 2010). Later, following the divergence of the two species, the soybean genome duplicated again ~13 MYA and, as a result, there are typically two soybean orthologues present for every common bean gene (Lin *et al*., 2010; Schmutz *et al.*, 2014). This trend is consistent with the present findings, where common bean contains approximately half the number of CLE peptide-encoding genes as soybean. The findings are also consistent with *Arabidopsis*, which is reported to have only 32 CLE peptide-encoding genes (Cock and McCormick, 2001), and is well known for fractionation (i.e. preferentially removing redundant and/or excess genomic information; Thomas *et al.*, 2006). Indeed, Group VI of the soybean and common bean CLE peptide families identified here is completely absent from *Arabidopsis*. This category is known to be induced by rhizobia to control legume nodulation (reviewed in Hastwell *et al.*, 2015), suggesting that either *Arabidopsis* has completely lost this group, or that the legume species have gained it as a means of regulating the relationship with their symbiotic partner.

Additional methods were employed here to identify conclusively soybean and common bean orthologues of a number of key CLE peptide-encoding genes of *Arabidopsis*. Indeed, orthologues of *AtCLV3*, which acts in the SAM to control stem cell numbers (Gaillochet *et al.*, 2015), were identified in common bean, and confirmed in soybean and *M. truncatula* (Chen *et al.*, 2009; Wong *et al.*, 2013). Interestingly, it is also shown that *MtCLV3* encodes three CLE peptide domains, but only one is translated due to the presence of an intron. Orthologues of *AtCLE40*, which acts in the RAM to control stem cell numbers (Hobe *et al.*, 2003; Sharma *et al.*, 2003; Stahl *et al.*, 2009), were also identified here in these same three legume species. This includes the homeologous copy of *GmCLE40a*, called *GmCLE40b*, which is unlikely to produce a functional product due to a naturally occurring mutation that truncates the pre-propeptide prior to the CLE domain. Orthologues of the three TDIF CLE peptide-encoding genes of *Arabidopsis*, which act throughout the plant in vascular differentiation (Grienenberger and Fletcher, 2015), were also identified here, including six genes in soybean, three in common bean, and three in *M. truncatula*. The predicted TDIF-encoding genes (together with one other soybean gene of unknown function) make up Group III of the CLE pre-propeptide family. A number of additional *Arabidopsis* orthologue candidates were also identified throughout the other various CLE peptide groups defined here.

Genome-wide searches to identify CLE peptide-encoding genes in legumes have been conducted previously using soybean, *M. truncatula*, and *L. japonicus* (Cock and McCormick, 2001; Oelkers *et al.*, 2008; Okamoto *et al.*, 2009; Mortier *et al.*, 2010, 2011; Lim *et al.* 2011), with a few additional genes also identified in common bean (Oelkers *et al.*, 2008; Ferguson *et al.*, 2014). However, many of these studies were limited by the technology and bioinformatic resources available at the time. Recent bioinformatic advances were capitalized on here to identify, and subsequently characterize, categorize, and compare thoroughly, the CLE peptide families of soybean and common bean. This also enabled unification of the nomenclature for these species, taking into account the duplicated nature of the soybean genome and the presence of orthologous genes amongst the two species.

Taken together, this research helped to assemble the complete CLE peptide families of two agriculturally important legume species, categorized them into groups to provide insight into their structure and function, identified key orthologues existing amongst them and *Arabidopsis*, and used transcriptional evidence to help elucidate their localization and activity. This represents one of the most in-depth studies conducted within and between any CLE peptide family to date. Future work to establish unequivocally the function of these critical peptides, identify their binding partners, and determine the precise structural modifications of their mature ligands is now needed to enhance further the understanding of these novel hormones in regulating plant development.

## Supplementary data

Supplementary data are available at *JXB* online.

Figure S1. Soybean and common bean pre-propeptide phylogenetic tree.

Figure S2. Hydrophobicity plot of the CLE pre-propeptides of soybean, common bean, and *Arabidopsis*.

Figure S3. Soybean, common bean, and *Arabidopsis* pre-propeptide phylogenetic tree.

Table S1. CLE peptide-encoding genes of soybean.

Table S2. Frequency (%) of amino acid residues in CLE pre-propeptides of soybean, common bean, and *Arabidopsis.*

Table S3. Soybean CLE peptide-encoding gene expression from transcriptome databases.

Table S4. Common bean CLE peptide-encoding gene expression from A Common Bean Gene Expression Atlas (Jamie *et al.* 2014).

Table S5. *Arabidopsis thaliana* CLE peptide-encoding gene expression.

Supplementary Data
