# Supplementary material for: Genome-wide annotation and characterization of CLAVATA/ESR (CLE) peptide hormones of soybean (Glycine max) and common bean (Phaseolus vulgaris), and their orthologues of Arabidopsis thaliana
Source: J Exp Bot. 2015 Jul 17;66(17):5271–87. doi: 10.1093/jxb/erv351 (PMC4526924; doi:10.1093/jxb/erv351)
Supplement: Supplementary Data [file supp_66_17_5271__index.html]

Genome-wide annotation and characterization of CLAVATA/ESR (CLE) peptide hormones of soybean (Glycine max) and common bean (Phaseolus vulgaris), and their orthologues of Arabidopsis thaliana — Supplementary Data 

# Genome-wide annotation and characterization of CLAVATA/ESR (CLE) peptide hormones of soybean (*Glycine max*) and common bean (*Phaseolus vulgaris*), and their orthologues of *Arabidopsis thaliana*

## Supplementary Data

Data files

- Supplementary Data - Supplementary Data
