# Supplementary material for: Genome-wide annotation and characterization of CLAVATA/ESR (CLE) peptide hormones of soybean (Glycine max) and common bean (Phaseolus vulgaris), and their orthologues of Arabidopsis thaliana
Source: J Exp Bot. 2015 Jul 17;66(17):5271–87. doi: 10.1093/jxb/erv351 (PMC4526924; doi:10.1093/jxb/erv351)
Supplement: Supplementary Data [file supp_erv351_Hastwell_et_al_Supp__Figures_and_Tables.pdf]

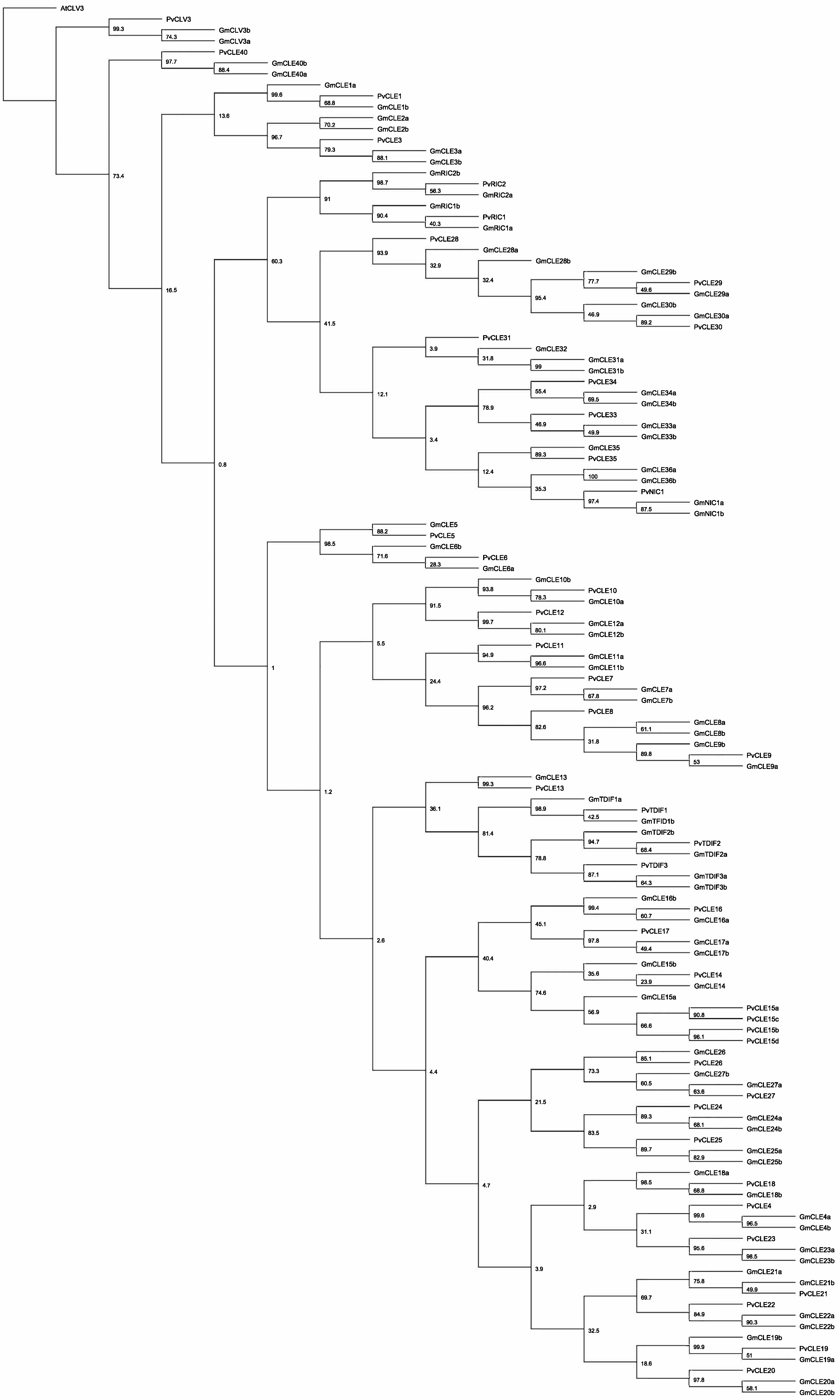
AtCLV3
PvCLV3
GmCLV3b
GmCLV3a
PvCLE40
GmCLE40b
GmCLE40a
GmCLE1a
PvCLE1
GmCLE1b
GmCLE2a
GmCLE2b
PvCLE3
GmCLE3a
GmCLE3b
GmRIC2b
PvRIC2
GmRIC2a
GmRIC1b
PvRIC1
GmRIC1a
PvCLE28
GmCLE28a
GmCLE28b
GmCLE29b
PvCLE29
GmCLE29a
GmCLE30b
GmCLE30a
PvCLE30
PvCLE31
GmCLE32
GmCLE31a
GmCLE31b
PvCLE34
GmCLE34a
GmCLE34b
PvCLE33
GmCLE33a
GmCLE33b
GmCLE35
PvCLE35
GmCLE36a
GmCLE36b
PvNIC1
GmNIC1a
GmNIC1b
GmCLE5
PvCLE5
GmCLE6b
PvCLE6
GmCLE6a
GmCLE10b
PvCLE10
GmCLE10a
PvCLE12
GmCLE12a
GmCLE12b
PvCLE11
GmCLE11a
GmCLE11b
PvCLE7
GmCLE7a
GmCLE7b
PvCLE8
GmCLE8a
GmCLE8b
GmCLE9b
PvCLE9
GmCLE9a
GmCLE13
PvCLE13
GmTDIF1a
PvTDIF1
GmTFID1b
GmTDIF2b
PvTDIF2
GmTDIF2a
PvTDIF3
GmTDIF3a
GmTDIF3b
GmCLE16b
PvCLE16
GmCLE16a
PvCLE17
GmCLE17a
GmCLE17b
GmCLE15b
PvCLE14
GmCLE14
GmCLE15a
PvCLE15a
PvCLE15c
PvCLE15b
PvCLE15d
GmCLE26
PvCLE26
GmCLE27b
GmCLE27a
PvCLE27
PvCLE24
GmCLE24a
GmCLE24b
PvCLE25
GmCLE25a
GmCLE25b
GmCLE18a
PvCLE18
GmCLE18b
PvCLE4
GmCLE4a
GmCLE4b
PvCLE23
GmCLE23a
GmCLE23b
GmCLE21a
GmCLE21b
PvCLE21
PvCLE22
GmCLE22a
GmCLE22b
GmCLE19b
PvCLE19
GmCLE19a
PvCLE20
GmCLE20a
GmCLE20b

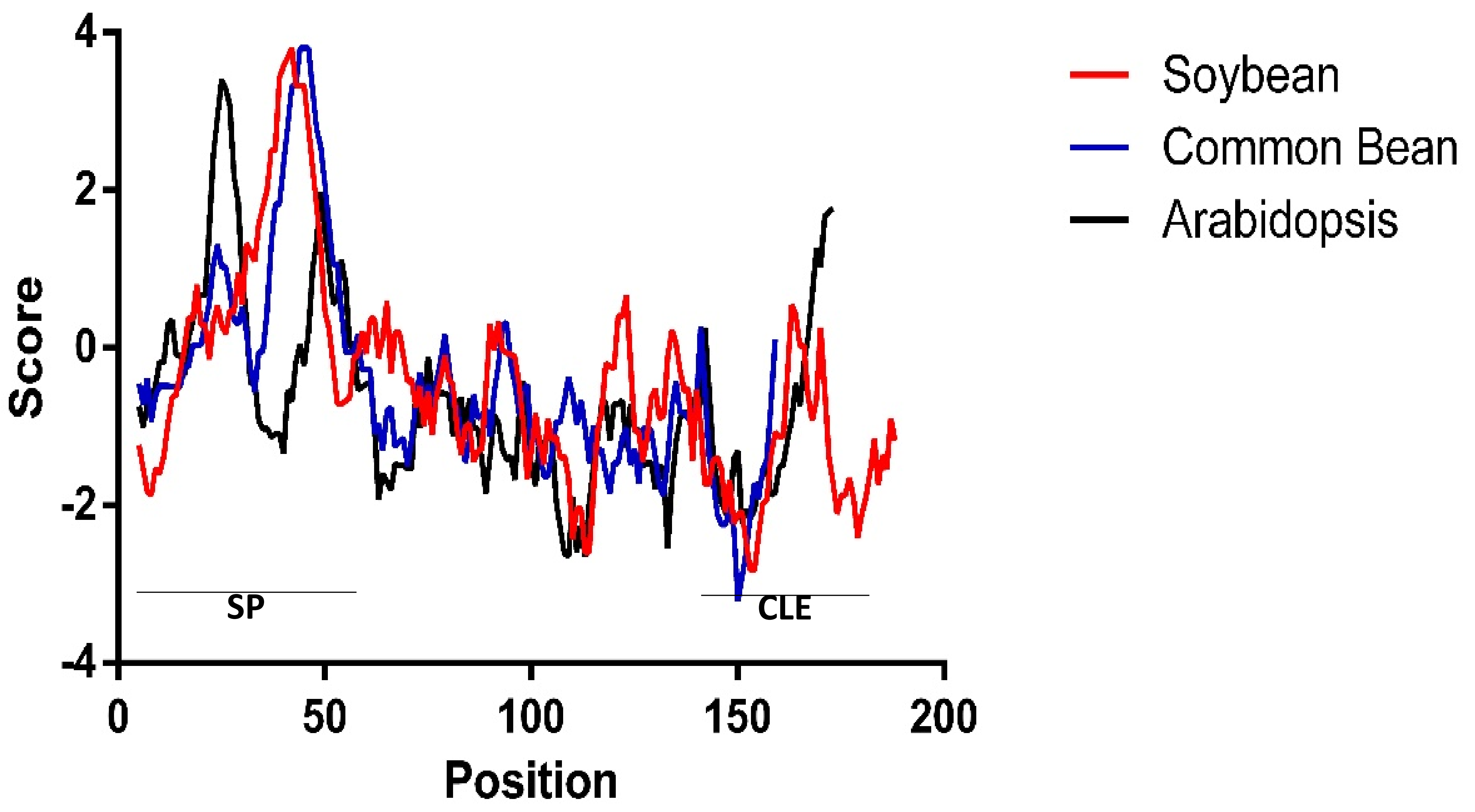

Supplementary Figure 2. Hydrophobicity plot of the CLE prepropeptides of soybean, common bean and Arabidopsis. Hydrophobicity scores were calculated according to Kyte and Doolittle (1982) for each amino acid in all Soybean (*G. max*), Common Bean (*P. vulgaris*), and Arabidopsis (*A. thaliana*) CLE prepropeptides based on the multiple sequence alignments shown in Figures 1 and 2. Amino acid hydrophobicity scores range from 4, being hydrophobic, to -4, being hydrophilic. Sequence positions highlighted include the hydrophobic Signal Peptide (SP) and CLE Domain (CLE).

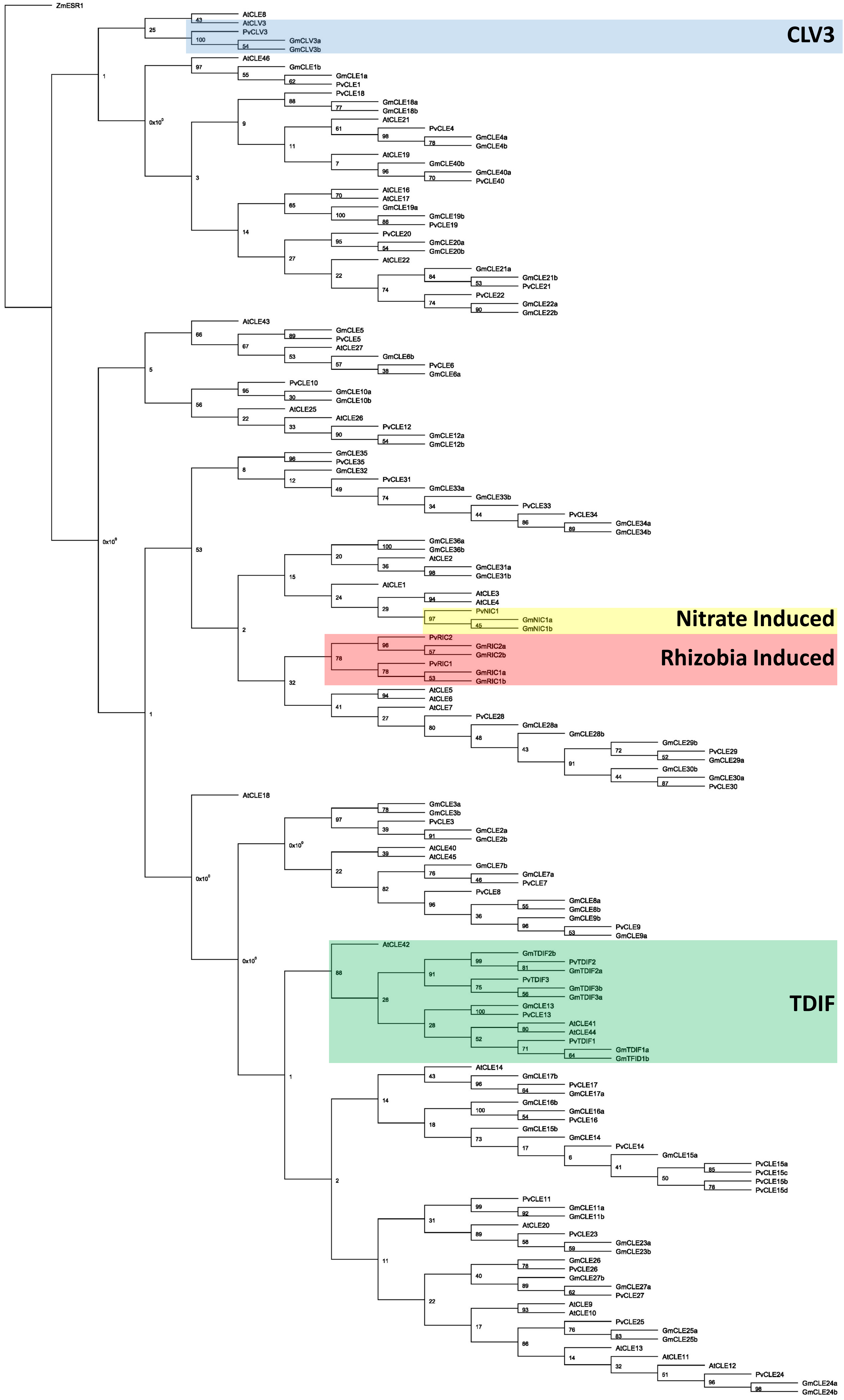

CLV3
Nitrate Induced
Rhizobia Induced
TDIF
ZmESR1
AtCLE8
AtCLV3
PvCLV3
GmCLV3a
GmCLV3b
AtCLE46
GmCLE1b
GmCLE1a
PvCLE1
PvCLE18
GmCLE18a
GmCLE18b
AtCLE21
PvCLE4
GmCLE4a
GmCLE4b
AtCLE19
GmCLE40b
GmCLE40a
PvCLE40
AtCLE16
AtCLE17
GmCLE19a
GmCLE19b
PvCLE19
PvCLE20
GmCLE20a
GmCLE20b
AtCLE22
GmCLE21a
GmCLE21b
PvCLE21
PvCLE22
GmCLE22a
GmCLE22b
AtCLE43
GmCLE5
PvCLE5
AtCLE27
GmCLE6b
PvCLE6
GmCLE6a
PvCLE10
GmCLE10a
GmCLE10b
AtCLE25
AtCLE26
PvCLE12
GmCLE12a
GmCLE12b
GmCLE35
PvCLE35
GmCLE32
PvCLE31
GmCLE33a
GmCLE33b
PvCLE33
PvCLE34
GmCLE34a
GmCLE34b
GmCLE36a
GmCLE36b
AtCLE2
GmCLE31a
GmCLE31b
AtCLE1
AtCLE3
AtCLE4
PvNIC1
GmNIC1a
GmNIC1b
PvRIC2
GmRIC2a
GmRIC2b
PvRIC1
GmRIC1a
GmRIC1b
AtCLE5
AtCLE6
AtCLE7
PvCLE28
GmCLE28a
GmCLE28b
GmCLE29b
PvCLE29
GmCLE29a
GmCLE30b
GmCLE30a
PvCLE30
AtCLE18
GmCLE3a
GmCLE3b
PvCLE3
GmCLE2a
GmCLE2b
AtCLE40
AtCLE45
GmCLE7b
GmCLE7a
PvCLE7
PvCLE8
GmCLE8a
GmCLE8b
GmCLE9b
PvCLE9
GmCLE9a
AtCLE42
GmTDIF2b
PvTDIF2
GmTDIF2a
PvTDIF3
GmTDIF3b
GmTDIF3a
GmCLE13
PvCLE13
AtCLE41
AtCLE44
PvTDIF1
GmTDIF1a
GmTFID1b
AtCLE14
GmCLE17b
PvCLE17
GmCLE17a
GmCLE16b
GmCLE16a
PvCLE16
GmCLE15b
GmCLE14
PvCLE14
GmCLE15a
PvCLE15a
PvCLE15c
PvCLE15b
PvCLE15d
PvCLE11
GmCLE11a
GmCLE11b
AtCLE20
PvCLE23
GmCLE23a
GmCLE23b
GmCLE26
PvCLE26
GmCLE27b
GmCLE27a
PvCLE27
AtCLE9
AtCLE10
PvCLE25
GmCLE25a
GmCLE25b
AtCLE13
AtCLE11
AtCLE12
PvCLE24
GmCLE24a
GmCLE24b

Supplementary Table 1. CLE peptide-encoding genes of soybean. Current and prior identifiers are listed, included those assigned here, and those previously used by Cock and McCormick (2001), Oelkers et al. (2008), Mortier et al. (2011), Lim et al. (2012) and Phytozome (v9.1 and earlier versions). Unannotated Phytozome genes are indicated by a location rather than an identifier.

Gene	Phytozome v10	Oelkers *et al.* 2008	Mortier *et al.* 2011	Lim et al. 2	uniprot.org	Phytozome v9.1*	Phytozome (earlier)
CLE30b	Chr06:36255159..36255402	-	-	-	-	Gm06:35,338,629..35,338,872	Gm06_31873
CLE30a	Chr06:36324860..36325095	-	-	-	-	Gm06:35,408,408..35,408,644	Gm06_31924
CLE33a	Chr06:36402219..36402452	-	-	-	-	Gm06:35,487,426..35,487,680	-
CLE14b	Chr10:46589943..46590137	-	-	GmCLE10-3	-	Gm10:46,018,999..46,019,250	Gm10_23522
CLE40b	Chr11:9961342..9961800	-	-	GmCLE11-1	-	Gm11:9,931,027..9,931,338	Gm11_4314
CLE22b	Chr12:27380684..27380911	-	-	GmCLE12-3	-	Gm12:20,718,769..20,718,996	Gm12_20250
CLE29a	Chr12:27615321..27615566	-	-	GmCLE12-2	-	Gm12:20,480,965..20,481,210	-
CLE28b	Chr12:38835186..38835383	-	-	-	-	Gm12:38,860,090..38,860,190	-
CLE34a	Chr12:38840660..38840902	-	-	GmCLE12-6	-	Gm12:38,865,464..38,865,706	Gm12_29982
CLE35	Chr13:28564185..28564418	-	-	GmCLE13-3	-	Gm13:27,370,526..27,370,834	-
CLE34b	Chr13:37353930..37354172	-	-	GmCLE13-6	-	Gm13:36,212,997..36,213,239	Gm13_23623
CLE10a	Glyma.01G038900	CLE34[#]	GmCLE02	Glyma01g04580	HM585100	Glyma01g04580	-
CLE7a	Glyma.01G047800	-	-	GmCLE01-2	-	Glyma01g05805	Gm01_2140
CLE27b	Glyma.01G056400	-	GmCLE19	Glyma01g06850	HM585117	Glyma01g06850	Gm01_54650
CLE4a	Glyma.01G196700	-	GmCLE33	-	HM585131	Glyma01g40455	Gm01_26914
CLE10b	Glyma.02G025700	-	GmCLE27	Glyma02g02980	HM585125	Glyma02g02980	Gm02_950
CLE7b	Glyma.02G107400	-	-	GmCLE02-2	-	Glyma02g11955	Gm02_47475
CLE27a	Glyma.02G115000	-	-	GmCLE02-3	-	Glyma02g12810	Gm02_47157
CLE23a	Glyma.02G270600	-	GmCLE22	Glyma02g43840	HM585120	Glyma02g43840	Gm02_27580
CLE21a	Glyma.02G278000	-	GmCLE18	Glyma02g44620	HM585116	Glyma02g44620	Gm02_24998
CLE20a	Glyma.03G125900	-	GmCLE38	GmCLE03-1	HM585136	Glyma03g28125	Gm03_18748
CLE3a	Glyma.03G238600	-	-	-	-	Glyma03g39855	Gm03_22730
CLE8b	Glyma.04G169400	-	-	Glyma04g33590	-	Glyma04g33590	Gm04_21157
CLE25a	Glyma.05G013600	-	GmCLE21	Glyma05g07900	HM585119	Glyma05g07900	Gm05_39153
CLE9a	Glyma.05G026700	-	-	-	-	Gm05:624,302..624,583	-
TDIF2a	Glyma.05G134700	-	GmCLE31	GmCLE05-2	HM585129	Glyma05g26513	Gm05_25424
CLE17a	Glyma.05G205400	-	GmCLE15	Glyma05g38040	HM585113	Glyma05g38040	Gm05_21288
CLE8a	Glyma.06G194000	-	-	Glyma06g20850	-	Glyma06g20850	Gm06_41985
CLE14	Glyma.06G221500	-	-	GmCLE06-2	-	Gm06:25,058,794..25,059,051	Gm06_37797
CLE29b	Glyma.06G231800	-	-	GmCLE06-4	-	Gm06:35,414,419..35,414,655	Gm06_31921
RIC2a	Glyma.06G284100	-	GmCLE35	GmRIC2	HM585133	Glyma06g43681	Gm06_26406
CLE31a	Glyma.07G204600	-	-	GmCLE07-1	-	Gm07:37,445,715..37,446,035	-
CLE19a	Glyma.07G219600	-	GmCLE28	Glyma07g34450	HM585126	Glyma07g34450	Gm07_19786
CLE22a	Glyma.07G235300	-	-	GmCLE07-3	-	Glyma07g36331	Gm07_23512
CLE17b	Glyma.08G012300	CLE63	GmCLE36	GmCLE08-1	HM585134	Glyma08g01531	Gm08_44735
TDIF2b	Glyma.08G089800	CLE51	GmCLE03	Glyma08g09500	HM585101	Glyma08g09500	Gm08_42343
CLE5	Glyma.08G262400	-	GmCLE26	GmCLE08-4	HM585124	Glyma08g31522	Gm08_32800
TDIF1a	Glyma.08G355400	-	-	Glyma08g47020	-	Glyma08g47020	Gm08_23134
TDIF3a	Glyma.09G048500	-	GmCLE29	Glyma09g05350	HM585127	Glyma09g05350	Gm09_1650
CLE16a	Glyma.09G140400	CLE57	GmCLE09	GmCLE09-2	HM585107	Glyma09g25983	Gm09_16903
CLE2b	Glyma.10G154900	-	GmCLE32	-	HM585130	Glyma10g29595	Gm10_20454
CLE24a	Glyma.10G205100	-	GmCLE24	Glyma10g34930	HM585122	Glyma10g34930	Gm10_22361
CLE15a	Glyma.10G237100	CLE58	GmCLE10	Glyma10g38160	HM585108	Glyma10g38160	Gm10_23520
CLE6b	Glyma.10G274200	-	GmCLE30	Glyma10g42070	HM585128	Glyma10g42070	Gm10_24702
CLE4b	Glyma.11G045000	-	GmCLE16	-	HM585114	Glyma11g04831	Gm11_36888
CLE1a	Glyma.11G140700	-	-	-	-	-	-
CLE40a	Glyma.12G054900	CLE61	GmCLE13	GmCLE12-8	HM585111	Glyma12g05873	-
CLE1b	Glyma.12G064200	-	-	-	-	-	-
RIC2b	Glyma.12G121800	-	GmCLE37	GmRIC2-1	HM585135	Glyma12g14241	Gm12_5809
CLV3a	Glyma.12G187800	-	-	GmCLE12-7	-	Glyma12g31336	
NIC1a	Glyma.12G208900	-	-	Glyma12g33660	-	Glyma12g33660	Gm12_18481
RIC1b	Glyma.12G209000	-	GmCLE39	GmCLE12-4	HM585137	Glyma12g33665	-
CLE12a	Glyma.13G067000	-	-	GmCLE13-2	-	Glyma13g04895	Gm13_37953
CLE18a	Glyma.13G103000	-	-	GmCLE13-1	-	Glyma13g00291	Gm13_40112
CLE32	Glyma.13G171400	-	-	-	-	Glyma13g24026	-
CLE31b	Glyma.13G171500	-	-	GMCLE13-4	-	Gm13:27,376,809..27,377,129	
CLE36a	Glyma.13G232300	-	-	-	-	Gm13:33,138,563..33,138,790	Gm13_24802
CLE13	Glyma.13G263500	-	-	Glyma13g33855	-	Glyma13g33855	Gm13_16736

Gene	Phytozome v10	Oelkers *et al.* 2008	Mortier *et al.* 2011	Lim et al. 2	uniprot.org	Phytozome v9.1*	Phytozome (earlier)
CLE28a	Glyma.13G271600	-	-	GmCLE13-5	-	Glyma13g34685	Gm13_23624
RIC1a	Glyma.13G292300	CLE62	GmCLE14	GmRIC1	HM585112	Glyma13g36831	Gm13_22918
NIC1b	Glyma.13G292400	-	-	GmCLE13-8	-	Gm13:38,047,909..38,048,152	-
CLV3b	Glyma.13G313400	-	-	GmCLE13-9	-	Glyma13g38975	-
CLE21b	Glyma.14G036500	-	GmCLE23	Glyma14g04100	HM585121	Glyma14g04100	Gm14_50653
CLE23b	Glyma.14G046400	CLE23[#]	GmCLE01	Glyma14g05080	HM585099	Glyma14g05080	Gm14_1542
CLE11a	Glyma.14G087000	CLE59	GmCLE11	Glyma14g09600	HM585109	Glyma14g09600	Gm14_48657
CLE36b	Glyma.15G080400	-	-	-	-	Gm15:6,143,084..6,143,314	Gm15_2583
TDIF3b	Glyma.15G155800	CLE60	GmCLE12	Glyma15g16720	HM585110	Glyma15g16720	Gm15_5414
CLE16b	Glyma.16G194300	-	-	GmCLE16-1	-	Glyma16g31896	Gm16_17018
CLE18b	Glyma.17G056000	-	-	-	-	Gm17:4,528,349..4,528,594	-
CLE9b	Glyma.17G100200	-	-	Glyma17g10850	-	Glyma17g10850	Gm17_37443
CLE25b	Glyma.17G121800	-	GmCLE20	Glyma17g13100	HM585118	Glyma17g13100	Gm17_4155
CLE11b	Glyma.17G237400	-	GmCLE34	Glyma17g35570	HM585132	Glyma17g35570	Gm17_19258
TDIF1b	Glyma.18G171800	CLE53	GmCLE05	Glyma18g37720	HM585103	Glyma18g37720	Gm18_39105
CLE12b	Glyma.19G017400	-	-	GmCLE19-1	-	Glyma19g02051	Gm19_51075
CLE20b	Glyma.19G128400	CLE54	GmCLE06	GmCLE19-2	HM585104	Glyma19g30865	Gm19_20810
CLE3b	Glyma.19G236000	-	-	-	-	Glyma19g42415	Gm19_24858
CLE19b	Glyma.20G017800	CLE56	GmCLE08	Glyma20g02180	HM585106	Glyma20g02191	Gm20_735
CLE26	Glyma.20G027000	-	GmCLE25	Glyma20g03290	HM585123	Glyma20g03290	Gm20_1302
CLE22b	Glyma.20G042900	-	-	GmCLE20-3	-	Glyma20g08353	Gm20_41593
CLE6a	Glyma.20G115800	CLE55	GmCLE07	Glyma20g24960	HM585105	Glyma20g24960	Gm20_28992
CLE24b	Glyma.20G185400	CLE52	GmCLE04	Glyma20g32640	HM585102	Glyma20g32640	Gm20_26402
CLE2a	Glyma.20G233400	-	GmCLE17	-	HM585115	Glyma20g37711	Gm20_24666
CLE37a	Glyma.16G047700	-	-	-	-	-	-
CLE37b	Glyma.19G103800	-	-	-	-	-	-

[#] Original identifiers used by Cock and McCormick (2001)

* Identifier also used by soybase.org

Supplementary Table 2. Frequency (%) of amino acid residues in CLE prepropeptides of soybean, common bean and Arabidopsis.

Amino Acid	*G. max* and *P. vulgaris*	*G. max*	*P. vulgaris*	*A. thaliana*
Ala	5.1	5.1	5.0	4.4
Cys	1.3	1.3	1.2	0.8
Asp	2.9	2.8	2.9	3.8
Glu	3.9	4.0	3.6	4.7
Phe	5.2	5.2	5.2	5.0
Gly	5.3	5.5	5.1	5.4
His	5.3	5.2	5.6	4.3
Ile	4.8	4.8	4.9	5.2
Lys	6.2	6.2	6.3	5.8
Leu	11.4	11.3	11.5	11.4
Met	2.9	2.9	3.0	3.1
Asn	5.3	5.4	5.2	4.6
Pro	6.6	6.5	6.8	7.0
Gln	3.4	3.3	3.5	2.6
Arg	6.3	6.4	6.0	8.5
Ser	11.3	11.2	11.4	10.3
Thr	5.1	5.0	5.3	4.9
Val	5.7	5.7	5.6	5.7
Trp	0.7	0.7	0.6	0.6
Tyr	1.3	1.3	1.4	2.0

Supplementary Table 3. Soybean CLE peptide-encoding gene expression from transcriptome databases.

Gene	Group	Root Hair 12HAI	Roothair_12HAImock	Root Hair 24 HAI	Roothair_24HAImock	Root Hair 48 HAI	Roothair_48HAImock	Root Hair 48 HAI Stripped	SAM	Green Pods	Root_tip	Flower	Leaves	Nodule	Root	one cm pod	pod shell 10DAF	pod shell 14DAF	seed 10DAF	seed 14DAF	seed 21DAF	seed 25DAF	seed 28DAF	seed 35DAF	seed 42DAF	Source
CLE1b	II	0	0	0	0	0	0	0.3	0	0	0	1.5	0	0	3.4											1
												0	0	1	7	0	0	0	0	0	0	0	0	0	0	2
CLE6a	II	0.8	2.5	3.6	2.2	1.8	2.9	2.1	0.5	0.7	2.5	1.2	2.8	1.1	3.2											1
												1	1	1	4	1	1	0	0	0	0	0	0	0	0	2
CLE6b	II	0.8	1.4	0.8	0.3	1.8	1.2	1.3	0.5	0.7	1.5	0.9	0.4	0.3	1.8											1
												3	1	0	3	0	3	2	0	0	0	0	0	0	0	2
CLE8a	II	0	0	0	0	0	0	0	0.3	0	0	0	0	0	0.3											1
												0	0	0	1	0	0	0	0	0	0	0	0	0	0	2
CLE8b	II	0	0	0	0	0.3	0.2	0.8	0	0	0	0	0	0	0.3											1
												0	0	2	0	1	1	0	0	0	1	0	0	0	1	2
CLE9b	II	0	0.4	0.3	0	0.3	0.2	0.3	0.5	0.7	0	0.3	0	0	1.1											1
												0	0	0	0	0	0	0	0	0	1	0	0	0	0	2
CLE10a	II	0.4	0	0.3	0	0.6	0.2	1.8	4.8	0.7	0.3	4.2	2.1	0.8	6.1											1
												3	2	0	4	1	0	1	1	1	1	1	1	1	1	2
CLE10b	II	0	0.4	0	0.3	0.3	0	1.3	0.8	1.4	0.3	5	1.8	0	2.6											1
												1	1	1	0	1	1	1	0	0	0	0	0	1	0	2
CLE11a	II	0.8	0	0.3	0	0	0	0	0.5	0	0	0	0	0.3	0.8											1
												0	0	1	2	0	0	0	1	0	0	0	0	0	0	2
CLE11b	II	0	0	0.6	0	0.6	0.7	0	0.8	0	0	0.3	0	1.6	2.1											1
												0	1	2	1	0	0	0	1	1	0	0	1	1	0	2
CLE17a	IV	2.8	3.9	0.8	1.9	1.5	4.4	2.1	0	0	0	0	0.4	0.5	1.8											1
												0	0	0	0	0	1	0	0	0	0	0	0	0	0	2
CLE19a	V	1.6	1.4	1.7	1.1	0.9	1	1.6	5.8	4.8	0.9	1.8	0.7	0	0.3											1
												8	24	0	1	16	13	11	1	2	5	1	0	1	0	2
CLE21a	V	0	0	0	0.3	0	0	0	1	0	0	0.3	0	0	0.5											1
												0	1	0	0	1	1	0	1	4	3	2	2	2	1	2
CLE21b	V	0	0	0	1.1	0.3	0.5	0.3	1.8	0	0	0	0	0	0.8											1
												1	3	0	1	1	0	1	0	1	3	4	3	11	4	2
CLE23a	V	2	0.4	1.4	1.4	0	0	0	0	0	0.9	0	1.1	0	0.8											1
												0	2	1	0	0	1	1	0	0	0	1	0	0	0	2
CLE23b	V	4	4.9	3.3	2.5	3.1	4.9	6.7	0.8	0.7	1.5	0.6	1.4	2.4	6.1											1
												1	0	2	6	2	3	5	2	3	1	2	1	2	0	2
CLE24a	V	0	0	0	0	0	0	0	0	0	0.3	0.3	0	0	0.3											1
												0	0	0	0	0	1	0	0	0	0	0	0	0	0	2
CLE24b	V	0.4	0	0.8	0.3	0.6	1.5	0.3	0	0	0.6	0	0	0	1.3											1
												0	0	0	2	0	0	0	0	0	0	0	0	0	0	2
CLE25a	V	13	9.5	9.2	17	8.9	10	0.3	0	0	6.8	0	0	0	4.5											1
												0	0	0	12	0	0	0	0	0	0	0	0	0	0	2
CLE25b	V	17	16.3	9.2	13.6	7.4	9.8	0.5	0	0	0	4	0	0.3	6.1											1
													0	1	6	0	0	0	0	0	0	0	0	0	0	2
CLE26	V	0	0	0	0.3	0	0	0	0	0	0	0.6	0	0	0											1
												0	2	0	0	0	1	0	0	0	3	2	1	1	1	2
CLE27a	V	0	0	0	0	0	0.2	0.3	0	0	0	0.6	1.1	0	0											1
												5	1	0	0	0	1	1	1	0	0	2	0	0	0	2
CLE27b	V	0	0	0	0	0	0	0	0.3	0	0	0	1.8	0	0											1
												4	5	0	0	1	2	1	1	1	3	0	0	0	1	2
TDIF2b	III	0.4	0	0	0.3	0	0	0.8	2.3	4.1	0	0	0	0.3	0.8											1
												4	4	0	0	9	11	11	4	3	3	2	1	1	0	2
TDIF1a	III	24	45	29	34	17	20	22	18	33	3.1	28	11	5.6	30											1
												29	16	5	9	16	13	10	1	2	4	3	2	2	2	2
TDIF1b	III	1.6	1.4	2.5	2.2	0.6	1.7	23	30	16	0	8.6	0.7	9.8	18											1
												13	6	4	6	11	9	9	1	2	2	3	1	3	0	2
TDIF3a	III	0	0	0	0	0	0	0.5	0	0.7	0	0.9	0	0	0.3											1
												3	3	0	0	0	1	0	4	3	3	2	0	1	0	2
TDIF3b	III	0	0	0	0	0	0	0.8	2	1.4	0	0.6	0	0	0											1
												4	1	0	0	1	1	0	0	0	0	1	0	0	0	2

1 Soybean eFP Browser (http://bar.utoronto.ca/efpsoybean/cgi-bin/efpWeb.cgi) with values presented as absolute RPKM (Reads/kilobase/million) expression. **2** Soybase RNA-Seq Atlas (http://www.soybase.org/soyseq/;)with values presented as normalised RPKM expression.

Supplementary Table 4. Common bean CLE peptide-encoding gene expression from A Common Bean Gene Expression Atlas (Jamie et al. 2014). Gene expression is displayed as normalised RPKM (Reads/ kilobase/million) values.

Gene	Grp	YL	L5	LF	LE	LI	YS	ST	FY	PY	PH	P1	P2	SH	S1	S2	RT	YR	R5	RF	RE	RI	N5	NE	NI
PvCLE3	II	0	0	1	1	2	2	0	0	0	1	0	2	0	0	0	0	0	0	0	0	0	0	0	0
PvCLE4	II	0	0	0	0	0	0	2	1	0	0	0	0	1	1	0	1	0	7	7	1	3	2	0	0
PvCLE5	II	1	0	1	0	0	0	0	1	0	3	0	0	0	0	0	0	0	0	0	0	0	1	0	0
PvCLE6	II	7	8	6	4	3	2	5	1	1	3	2	3	0	2	2	21	24	21	13	13	12	12	5	8
PvCLE7	II	3	2	1	1	1	1	3	2	1	2	1	1	1	1	0	1	3	3	3	1	1	1	1	1
PvCLE8	II	1	0	0	0	0	1	2	0	0	0	0	0	0	0	1	0	0	1	0	0	0	0	0	0
PvCLE9	II	0	0	0	0	0	0	0	0	0	0	0	0	0	0	0	0	0	0	0	0	0	0	0	0
PvCLE10	II	5	7	9	6	13	26	9	9	1	6	4	21	9	2	5	3	7	15	20	8	12	3	3	18
PvCLE11	II	5	2	1	0	0	10	18	2	0	2	1	1	1	1	0	3	2	6	5	5	7	27	13	14
PvCLE12	II	2	1	0	0	0	9	8	4	0	0	0	0	3	3	2	1	2	9	8	3	3	0	0	0
PvCLE14	IV	0	0	0	0	0	0	0	0	0	0	0	0	0	0	0	0	0	1	1	1	1	0	0	1
PvCLE16	IV	7	6	29	12	10	6	13	30	13	29	14	39	2	2	1	0	2	3	2	3	3	1	1	15
PvCLE17	IV	1	3	6	5	2	6	1	0	0	0	3	6	0	0	0	0	5	2	2	2	5	2	3	4
PvCLE18	V	0	0	0	0	0	0	0	0	0	0	0	0	0	0	0	0	0	0	0	0	0	0	0	0
PvCLE19	V	14	5	6	3	3	1	7	6	1	3	1	2	12	4	2	11	3	3	4	5	2	10	0	0
PvCLE20	V	3	1	1	0	1	24	10	5	0	0	0	0	3	2	1	0	2	2	2	4	10	2	1	0
PvCLE21	V	0	0	0	0	0	0	0	0	0	0	0	0	1	0	0	0	0	0	0	0	0	0	0	0
PvCLE22	V	0	0	0	0	0	0	0	0	0	0	0	0	0	0	0	0	0	0	0	0	0	0	0	0
PvCLE23	V	26	58	3	8	35	25	11	1	2	43	5	7	6	1	1	7	57	59	6	33	67	28	11	28
PvCLE24	V	1	1	1	1	0	5	0	0	0	1	0	0	0	0	0	0	0	1	1	1	1	0	0	0
PvCLE25	V	0	1	0	0	0	0	2	1	0	0	0	0	2	5	6	42	55	30	25	25	13	21	6	63
PvCLE26	V	23	32	14	11	14	7	10	10	5	16	3	4	44	23	17	1	0	2	0	2	2	0	0	0
PvCLE27	V	5	3	4	4	5	0	0	2	0	0	1	5	1	1	0	0	0	0	0	1	0	0	0	0
PvCLE28	VII	0	0	0	0	0	0	0	0	0	0	0	0	0	0	0	2	6	5	1	1	0	0	0	0
PvCLE29	VII	0	0	0	0	0	0	0	160	0	0	0	0	0	0	0	0	0	0	0	0	0	0	0	0
PvCLE40	I	0	0	0	0	0	0	0	0	0	0	0	0	0	0	0	0	0	0	0	0	0	0	0	0
PvCLV3	I	0	0	0	0	0	0	0	0	0	0	0	0	0	0	0	0	0	0	0	0	0	0	0	0
PvNIC1	VII	0	0	0	0	0	0	0	0	0	0	0	0	0	0	0	0	1	4	4	1	0	9	11	0
PvRIC1	VI	0	0	0	0	0	0	0	0	0	0	0	0	0	0	0	0	1	1	0	2	1	21	0	0
PvRIC2	VI	0	2	0	0	0	0	0	0	0	0	0	0	0	0	0	0	0	0	3	19	21	163	5	11
PvTDIF1	III	21	11	10	9	79	72	35	25	7	35	15	46	9	16	7	6	12	43	25	20	51	30	11	34
PvTDIF2	III	1	1	1	1	0	4	5	4	2	16	5	13	4	1	1	0	1	1	1	1	1	0	0	0
PvTDIF3	III	0	0	0	0	0	0	2	1	1	3	1	1	3	1	0	0	0	0	0	0	0	0	0	0

Tissue type key:

YL- Fully expanded 2nd trifoliate leaf tissue from plants provided with fertiliser
L5- Leaf tissue collected 5 days after plants were inoculated with effective rhizoubium
LF- Leaf tissue from fertilised plants collected at the same time of LE and LI
LE- Leaf tissue collected 21 days after plants were inoculated with effective rhizobium
LI- Leaf tissue collected 21 days after plants were inoculated with ineffective rhizobium
YS- All stem internodes above the cotyledon collected at the 2nd trifoliate stage
ST- Shoot tip, including the apical meristem, collected at the 2nd trifoliate stage
FY- Young flowers, collected prior to floral emergence
PY- Young pods, collected 1 to 4 days after floral senescence. Samples contain developing embryos at globular stage
PH- Pods approximately 9cm long, associated with seeds at heart stage (pod only)
P1- Pods between 10 and 11 cm long, associated with stage 1 seeds (pod only)
P2- Pods between 12 and 13 cm long associated with stage 2 seeds (pod only)
SH- Heart stage seeds, between 3 and 4 mm across and approximately 7 mg
S1- Stage 1 seeds, between 6 and 7 mm across and approximately 50 mg
S2- Stage 2 seeds, between 8 and 10 mm across and between 140 and 150 mg
RT- Root tips, 0.5 cm of tissue, collected from fertilized plants at 2nd trifoliate stage of development.
YR- Whole roots, including root tips, collected at the 2nd trifoliate stage of development
R5- Whole roots separated from 5 day old pre-fixing nodules
RF- Whole roots from fertilized plants collected at the same time as RE and RI
RE- Whole roots separated from fix+ nodules collected 21 days after inoculation
RI- Whole roots separated from fix- nodules collected 21 days after inoculation
N5- Pre-fixing (effective) nodules collected 5 days after inoculation
NE- Effectively fixing nodules collected 21 days after inoculation
NI- Ineffectively fixing nodules collected 21 days after inoculation

Supplementary Table 5. *Arabidopsis thaliana* CLE peptide-encoding gene expression. Gene expression is displayed as normalised log2-transformed values (4.1 = 17 fold).

Research Area	Dev	Dev	Dev	Dev	Dev	Dev	Dev	Dev	Dev	Dev	Dev	Dev	Poll	Sd+Sq	Sd+Sq	PhCh	PhCh	PhCh	CAGE	CAGE	CAGE	MS	MS	MS	MS	MS	MS	MS	MS
Growth Stage	1	1	1.09	1.11	1.12	6.1	1.1	1.09	1	1	1	1.04	6.5	9.7	9.7	1	1.03	1.09	1.03	3.7	1.03	1.09	1.09	1	1	1	1	1.09	1.09
Tissue	Cotyl	Hyptl	FlwRos	FlwRos	FlwRos	SenLf	CaulLf	ShoApx.Infl	Root	ShoApx.Veg	Sdlng.grn	Root	Mat.Poll	Seed3+Sil	Seed10-Sil	VegRos	VegRos	VegRos	Lf	Flw	Root	Sdlng.grn	Sdlng.grn	Root	Root	Sdlng.grn	Sdlng.grn	Root	Root
AtCLE12	0.1	0.7	-0.2	0	0	0	0.1	-0.5	0.3	-0.1	0	0.2	0.7	-0.2	0	0	-0.1	-0.1	0	-0.5	1.8	-0.4	-0.3	0.8	1.8	-0.1	-0.3	1.3	1.6
AtCLE3	0	0	0	0	0	0	0	0	0.3	0	0	0.4	0.4	0	0.1	0	0	0	0	0	0	0	0	0.2	0	0	0	0	0
AtCLE6	0	1.2	-0.1	-0.1	0	-0.1	0	0	3.6	0	0	3.7	0.3	0.1	0.9	0	0	0	0	-0.2	2.6	0	0.2	1.8	1.5	0	0	1.3	0.8
AtCLE2	0.2	0	0	0	0.1	0	0	-0.1	4.1	0	0	3.9	0.6	-0.1	0.4	0.1	0	0	0.1	-0.1	3.4	0	-0.1	2.7	2.7	0	0	2.6	2.3
AtCLE17	-0.1	0.4	-0.1	-0.1	-0.2	-0.8	-0.3	0.7	0.4	0.6	0	0	0	0.1	-0.4	0.1	0.1	0	0.1	0.2	0.3	0.2	0	0.2	0.1	0.3	0.3	0.3	0.1
AtCLE27	-0.2	0.1	0	0.1	0.1	-0.4	-0.2	1.1	-0.1	0.6	-0.1	0	0.4	0	0.1	0	0.2	0.3	0	0.4	0.3	-0.1	-0.4	-0.5	-0.1	-0.2	-0.2	-0.3	-0.1
AtCLE46	0	1.5	0.7	0.7	0.6	-0.2	0	1.9	0.9	0.2	0	2.1	0.4	0.3	0	0	-0.1	0	0	0.5	0	0.2	0.2	-0.2	0	0	0	-0.1	-0.1
AtCLE41	0	1.7	0.3	0.4	0	-1.6	-0.8	0.5	0.2	1	0.2	0.1	-1.8	0.1	-2.1	0	0	0	-0.2	0	-0.3	-0.7	-0.6	-1	-0.6	-0.8	-0.4	-0.3	-0.2
AtCLE44	0	1.6	0.2	0.5	0.4	0.8	0	0.6	0.1	0.4	0.1	-0.2	-0.5	-0.2	-0.9	0	-0.2	-0.1	0.5	0	0	0	0.5	-0.4	-0.7	-0.4	0.1	1.1	1
AtCLE9	0	-0.6	0.2	0.2	0.2	-0.2	0.1	-0.1	-0.5	-0.2	0.5	-0.4	0.4	0	0.1	0.7	0.8	0.7	0.6	0.1	0	0.4	0.2	-0.2	-0.2	0.5	0.5	-0.3	-0.4
AtCLV3	0	-0.1	0	0	0	-0.1	0	0.1	-0.1	0.4	0	-0.2	1.8	-0.1	0.9	0	-0.2	0	0.1	-0.3	-0.1	0	0	-0.2	-0.1	0	0	0	-0.1
AtCLE40	0	-0.1	0	0	0	0	0	0	-0.1	0	0	-0.1	0.6	0	0.3	0	0	0	0	-0.1	0	0	0	0	0	0	0	0	0
AtCLE26	0	1.2	-0.1	0	0.3	-0.1	1.5	-0.1	0.1	0	0	0.2	0.6	1.3	-0.2	0.4	-0.1	-0.1	0.2	0.4	-0.1	0.5	0.4	-0.1	-0.1	0.3	0.1	0	0.2
AtCLE21	0.2	0.3	0.1	0.1	0.3	0.7	0.7	0.1	-0.1	0	0	-0.1	0.1	0.1	0	0	0	0	0	-0.1	-0.3	0	0	-0.2	0	-0.1	0	-0.2	-0.1

Tissue type key:

Cotyl	Cotyledon
Hyptl	Hypocotyl
FlwRos	Entire Rosette After Transitioning to Flowering
SenLf	Senescing Leaf
CaulLf	Cauline Leaf
ShoApx.Infl	Shoot Apex, Infloresence
Root	Root
ShoApx.Veg	Shoot Apex, Vegetative
Sdlng.grn	Seedling, Green Parts
Root	Root
Mat.Poll	Mature Pollen
Seed3+Sil	Seeds Stage 3 w/o Siliques
Seed10-Sil	Seed Stage 10 w/ Siliques
VegRos	Vegetative Rosette
Lf	Leaf
Flw	Flower

Dev	Developmental Baseline
Poll	Pollen
Sd+Sq	Seed and Silique Development
PhCh	Phase Change
CAGE	Comparison with CAGE (Compendium of Arabidopsis Gene Expression)
MS	Development on MS Agar
